# Provable Boolean interaction recovery from tree ensemble obtained via random forests

**DOI:** 10.1073/pnas.2118636119

**Published:** 2022-05-24

**Authors:** Merle Behr, Yu Wang, Xiao Li, Bin Yu

**Affiliations:** ^a^Department of Statistics, University of California, Berkeley, CA 94720;; ^b^Department of Electrical Engineering and Computer Sciences, University of California, Berkeley, CA 94720;; ^c^Center for Computational Biology, University of California, Berkeley, CA 94720

**Keywords:** decision trees, interaction selection, ensemble methods, consistency, interpretable machine learning

## Abstract

Random Forests (RFs) are at the cutting edge of supervised machine learning in terms of prediction performance, especially in genomics. Iterative RFs (iRFs) use a tree ensemble from iteratively modified RFs to obtain predictive and stable nonlinear or Boolean interactions of features. They have shown great promise for Boolean biological interaction discovery that is central to advancing functional genomics and precision medicine. However, theoretical studies into how tree-based methods discover Boolean feature interactions are missing. Inspired by the thresholding behavior in many biological processes, we first introduce a discontinuous nonlinear regression model, called the “Locally Spiky Sparse” (LSS) model. Specifically, the LSS model assumes that the regression function is a linear combination of piecewise constant Boolean interaction terms. Given an RF tree ensemble, we define a quantity called “Depth-Weighted Prevalence” (DWP) for a set of signed features $S^{\pm }$. Intuitively speaking, DWP($S^{\pm }$) measures how frequently features in $S^{\pm }$ appear together in an RF tree ensemble. We prove that, with high probability, DWP($S^{\pm }$) attains a universal upper bound that does not involve any model coefficients, if and only if $S^{\pm }$ corresponds to a union of Boolean interactions under the LSS model. Consequentially, we show that a theoretically tractable version of the iRF procedure, called LSSFind, yields consistent interaction discovery under the LSS model as the sample size goes to infinity. Finally, simulation results show that LSSFind recovers the interactions under the LSS model, even when some assumptions are violated.

## Significance

Random Forests (RFs) are among the most successful machine-learning algorithms in terms of prediction accuracy. In many domain problems, however, the primary goal is not prediction, but to understand the data-generation process—in particular, finding important features and feature interactions. There exists strong empirical evidence that RF-based methods—in particular, iterative RF (iRF)—are very successful in terms of detecting feature interactions. In this work, we propose a biologically motivated, Boolean interaction model. Using this model, we complement the existing empirical evidence with theoretical evidence for the ability of iRF-type methods to select desirable interactions. Our theoretical analysis also yields deeper insights into the general interaction selection mechanism of decision-tree algorithms and the importance of feature subsampling.

Supervised machine learning (ML) algorithms have been proven to be extremely powerful in a wide range of predictive tasks from genomics to cosmology to pharmacology. Understanding how a model makes predictions is of paramount value in science and business alike (1). For example, when a geneticist wants to understand a particular disease—e.g., breast cancer—a black-box algorithm predicting the risk of breast cancer from genotype features is useful, but it does not offer biological insight.

That is, discovery of genes and gene interactions driving a particular disease provides not only understanding as a basic goal in science, but also opens doors for therapeutic treatments. It is a pressing task, in genomics and beyond, to interpret supervised ML models or algorithms and extract mechanistic information in addition to prediction.

Among many supervised ML algorithms, tree ensembles, such as those from Random Forests (RFs) (2) and gradient-boosted decision trees (3), stand out, as they enjoy both state-of-the-art prediction performance in a variety of practical problems and lead to relatively simple interpretations (4–8). To interpret a tree ensemble model, two questions are central:

• **Feature importance**: What features are important for the model’s prediction?
• **Interaction importance**: What interactions among features are important for the model’s prediction?

While many studies (refs. 4 and 6–8 and the references therein) focus on the RF feature importance, there are relatively few results on the second question. In genetics, Wan et al. (9) and Yoshida and Koike (10) seek (higher-order) gene interactions (or epistasis) by extracting genetic variant interactions from paths of ensembles of fitted decision trees. Wan et al. (9) use MegaSNPHunter based on boosting trees and interpret all groups of features that jointly appear on one of the decision paths as a candidate interaction. Yoshida and Koike (10) propose to rank interactions of genetic variants based on how often they appear together on decision paths in an RF tree ensemble. Recently, iterative RFs (iRFs) (11) were proposed to seek predictive, stable, and high-order nonlinear or Boolean feature interactions. Even though iRF uses the idea that the set of interacting features often appear together on individual decision paths of a tree in an RF ensemble, as in Yoshida and Koike (10), it uses several other ideas. That is, iRF incorporates a soft dimension-reduction step via iterative reweighting of features in terms of their Gini importance, in order to stabilize individual decision paths in the trees. Using the random intersection trees (RIT) (12) algorithm, iRF extracts stable interactions of arbitrary order in a computationally efficient way, even when the number of features is large. There is very positive evidence that iRF extracts predictive, stable, and high-order Boolean interaction information from RF in genomics and other fields (11, 13, 14). While all the works mentioned above provide strong empirical evidence that interactions extracted from the ensemble of decision trees via RF or iRF are informative about underlying biological functional relationships, there are no theoretical results regarding interaction discovery using RF, iRF, or other tree-based methods. In this paper, as a first step toward understanding the interaction-discovery property of tree-based methods, we investigate a key idea in the previous works (9–11)—namely, that frequent joint appearance of features on decision paths in the RF tree ensemble suggests an interaction.

One of the most common assumptions made in previous theoretical analyses of RF is a family of smoothness conditions on the underlying mean regression function, such as the Lipschitz smoothness condition (see, e.g., refs. 15–17). However, many biological processes show thresholding or discontinuous interacting behavior among biomolecules (18, 19), which strongly violates the Lipschitz assumption. It is therefore necessary to introduce a model that can capture the thresholding behavior through discontinuous mean regression function.

## The Locally Spiky Sparse Model.

Motivated by this thresholding behavior of biomolecules and inspired by RF’s predictive performance successes in genomics data problems (20–22), we consider the locally spiky sparse (LSS) model:* an additive regression model where the mean regression function is assumed to be a linear combination of Boolean interaction functions. The linear coefficients, as well as the threshold coefficients of the Boolean functions, are called “model coefficients.” Via Boolean functions, the LSS model is able to capture discontinuous thresholding behavior in biology; hence, it can be more relevant for biologists than models with smoothness constraints. We believe the LSS model is suitable and useful as a benchmark model under which to evaluate theoretically (and computationally) interaction-discovery performance of tree-based ML algorithms, including RF.

## Our Contributions.

Assume that independent and identically distributed (i.i.d.) data samples from the LSS model are given and an RF is fit to these data.

1. For an RF tree ensemble, we first define “signed features.” For a decision path of a set of signed features $S^{\pm }$ in the ensemble, we then define a quantity called “depth-weighted prevalence” (DWP). Intuitively speaking, DWP of $S^{\pm }$ measures how frequently the features in $S^{\pm }$ appear together in an RF tree ensemble. We show that DWP has a universal upper bound that depends only on the size of the set of signed features. Moreover, the upper bound is attained with high probability as the sample size increases if and only if the signed features represent a union of interactions in the LSS model. Based on DWP, we show that a simple algorithm—i.e., LSSFind, defined in Algorithm 1—can consistently recover interaction components in the LSS model, regardless of the model coefficients.
2. Our theoretical results imply that feature subsampling of RF is essential to recover interactions by the RF tree ensemble. When too few features are sampled at each node, the tree ensemble is close to extremely randomized trees, and DWP of any set of signed features is independent of the response, which means that it does not contain information on the LLS model; when too many features are sampled, all the trees in the ensemble will be very similar to one another, and that turns out to make it difficult to use tree structures to distinguish between interactions and noninteractions. More specifically, the ratio between the number of subsampled features *m_try_* and the total number of features *p* should be a nonzero constant in order for our algorithm to learn higher-order interactions from tree paths.

## Existing Theoretical Works on RF.

Existing theoretical studies of RF and its variants belong to two categories. The first focuses on estimating the regression function under Lipschitz or related conditions on the underlying regression function via averaging the decision trees in the RF tree ensemble. The second category studies feature importance measures as an RF output. In contrast, we provide a study on feature interaction selection consistency under an LSS model using DWP extracted from the RF tree ensemble.

In particular, in the first category, Biau (15) considers “median forests” (23), originally considered as a theoretical surrogate by Breiman (24), and obtains the *L*_2_ convergence rate under the Lipschitz continuous models. Scornet et al. (16) give the first consistency result for Breiman’s original RF with subsampling instead of bootstrapping in the low-dimensional setting when data are generated via an additive regression model with continuous components. Wager and Athey (17) consider a variant of RF, called honest RF, in the causal inference setup and prove its point-wise consistency and asymptotic normality when the conditional mean function is Lipschitz continuous. Similarly, Mentch and Hooker (25) showed that, under some Lipschitz-type conditions, a moderately large number of trees approximate well the infinite number of trees. Based on these asymptotic normality results, ref. 26 derived hypothesis tests for the null hypothesis that the regression function is additive. Thus, if one defines features interaction as the deviation from a continuous additive regression function, then their results enable testing on a particular candidate. In contrast, in this work, we define feature interaction via the noncontinuous Boolean functions in the LSS model, and we derive consistent interaction selection via the RF tree ensemble, as opposed to a test for an individual interaction, as in ref. 26.

The second category focuses on theory regarding individual feature importance measures. Results in this line of work do not rely on Lipschitz conditions. However, to the best of our knowledge, these works study statistical properties of only noisy features, but do not provide results for signal features in finite samples. Louppe et al. (5) show that Mean Decrease in Impurity (MDI) feature importance for randomized trees has a closed-form formula with an infinite number of samples. Zhou and Hooker (6) use out-of-sample data to improve the MDI feature importance with unbiased theoretical guarantees. Li et al. (8) show that the MDI feature importance of noisy features is inversely proportional to the minimum leaf-node size and suggest a way to improve the MDI using out-of-bag samples. Loecher (7) gives a family of MDI feature importance via out-of-bag samples that are unbiased for the noisy features. Moreover, many studies focus on permutation-based feature importance measures—in particular, Shapley effects (27–33). Among these works, ref. 33 shows some conceptual similarities to the DWP approach considered in this paper, as the authors also consider the concept of joint appearance of features on decision paths in the RF tree ensemble. However, instead of using this concept to extract feature interactions, as done in this work, they use it to define an importance sampling scheme to estimate the Shapley effects.

Also related to our work is the recent work ref. 34, which analyzes the extraction of rule sets from an RF tree ensemble. This is very similar to interaction selection, as considered in this work, except that the extracted rules in ref. 34 also include specific estimated thresholds for the individual features. The theoretical analysis in ref. 34 focuses on the stability of the selected rules without specifying a particular data-generating model. In contrast, this paper obtains model-selection-consistency results for LSSFind to estimate signed interactions of signal features under the LSS model.

The rest of the paper is organized as follows: Section 1 introduces the LSS model and Boolean interactions in more detail. Section 2 reviews the RF algorithm and formally defines DWP for a given set of signed features relative to an RF tree ensemble. Section 3 presents our main theoretical results for DWP and introduces LSSFind, a theoretically inspired algorithm to detect interactions from RF tree ensembles via DWP. Section 4 contains simulation results. We conclude with a discussion in Section 5.

## 1. LSS Model to Describe Boolean Interactions

In this section, we introduce necessary notations and a precise mathematical definition of the LSS model. To this end, for an integer N∈N, let [N]≔{1,2,…,N}. For a set *S* of finite elements of [N], let |S| denote its cardinality or the number of elements in *S*. For any event *A*, let 1(A) denote the indicator function of *A*. We assume a given dataset $D=\{(x_{1},y_{1}),\ldots ,(x_{n},y_{n})\}$ of *n* samples, with $x_{i}=(x_{i1},\ldots ,x_{in})\in R^{p}$ and $y_{i}\in R$. We say that the data D are generated from an LSS model when the following assumptions hold true.

LSS Model 1. *Assume* $D=\{(x_{1},y_{1}),\ldots ,(x_{n},y_{n})\}$ *are i.i.d. samples from a distribution P*(*X*,  *Y*), *such that for some fixed constants* $C_{\beta }>0,C_{\gamma }\in (0,0.5)$, *the regression function takes the following form*:

[1] $$E(Y|X)=\beta_{0}+\sum_{j=1}^{J}\beta_{j}\prod_{k\in S_{j}}1(X_{k}⋛\gamma_{k}),$$

*where* ⋛ *in* *Eq.* **1** *means either* ≤ *or* ≥, *potentially different for every k. Coefficients β_j_ are bounded from below, i.e.,*

[2] $$\overset{J}{\underset{j=1}{\min}}|\beta_{j}|>C_{\beta },$$

*and thresholds γ_j_ are bounded away from* 0 *and* 1*, i.e.,*

[3] $$\gamma_{j}\in (C_{\gamma },1-C_{\gamma }),$$

*for* j=1,…,J. $S_{1},\ldots ,S_{J}\subset [p]$ *are sets of features called basic interactions*. *We associate* ≤ *in* *Eq.* **1** *with a negative sign* (–1) *and* ≥ *with a positive sign* (+ 1), *such that a signed feature can be written as a tuple* $\left(k,b_{k})\in [p]\times \{-1,+1\right\}$. *We call* $S_{1}^{\pm },\ldots ,S_{J}^{\pm }\subset [p]\times \{-1,+1\}$ *basic signed interactions with* $S_{j}^{\pm }=\{(k,b_{k}):k\in S_{j}\}$.

Note that for interactions with only one feature *k*, due to the sign ambiguity in the LSS model—i.e., $1(X_{k}\leq a)=1-1(X_{k}>a)$—both {(k,−1)} and {(k,+1)} are counted as an interaction.

The LSS model aims to capture interactive thresholding behavior, which has been observed for various biological processes (18, 35–39). For example, in gene regulatory networks, often a few different expression patterns are possible. Switching between those patterns can be associated with individual components that interact via a threshold effect (36–38). Such a threshold behavior is also observed for other signal-transduction mechanisms in cells—e.g., protein kinase (35) and cell differentiation (18). Another example of a well-studied threshold effect is gene-expression regulation via small RNA (39). Although for most biological processes, the precise functional mechanisms between different features and a response variable of interest are much more complicated than what the LSS model can capture, theoretical investigations of a particular learning algorithm, such as RF, are only feasible within a well-defined and relatively simple mathematical model and useful for practice when such a model is empirically relevant. Given the empirically observed interactive threshold effects in many real biological systems, the LSS model clearly provides a useful enrichment to the current state of theoretical studies of RF and related methods, since current theoretical models do not capture the often-observed interactive threshold behavior.

In order to prove our main Theorem 2, we further impose the following constraints on the LSS model.

Constraint 1 **(C1)** (Uniformity): *X* is uniformly distributed on ${\left[0,1\right]}^{p}$.

This uniformity assumption implies that each feature is independent of each other. Because any decision tree remains invariant under any strictly monotone transform of an individual feature, the uniform distribution assumption of *X* can be relaxed to the assumption that individual features *X_j_*, j∈[p] are independent with a distribution that has Lebesgue density. We note that such an independence assumption might be violated in real-world problems. For example, for genetic data with single-nucleotide polymorphisms or gene expression as features *X_j_*, there will typically be a strong correlation between features that are located close by on the chromosome. However, in many cases, it is feasible to restrict to a subset of features (e.g., those that are located sufficiently far apart on the genome) in order to obtain approximate independence. In Section 4, we also demonstrate in simulations that for sufficiently weak feature correlation, one can still obtain accurate interaction selection with LSSFind.

**C2** (Bounded-Response): *Y* is bounded—i.e., |Y|<1.

Note that although we assume |Y|<1, the constant one can be changed to any constant, as we can scale *Y* by any positive number, and the conclusions in our main results will remain intact. This boundedness condition can be further relaxed so that the residue Z≔Y−E(Y|X) is independent of *X* and 1-subgaussian if we assume a slightly stronger assumption on *p* and *n* than the conditions in C4. See *SI Appendix*, Proposition S5 for more detail.

**C3** (Nonoverlapping Basic Interactions): $S_{1},\ldots ,S_{J}$ do not overlap—i.e., $S_{j_{1}}\cap S_{j_{2}}=\emptyset \text{for}\;\text{all}j_{1}\neq j_{2}.$

The nonoverlapping assumption that different interactions $S_{j_{1}},S_{j_{2}}$ with $j_{1}\neq j_{2}$ are disjoint might not always be justified in real-world problems. However, it is a crucial assumption for our theorem to hold. The general problem with overlapping interactions in the LSS model is that such models can be nonidentifiable, meaning that different forms of Eq. **1** can imply the same regression function E(Y|X). For example, for the response $1(X_{1}<0.5,X_{2}<0.5)+1(X_{1}>0.5,X_{2}>0.5)$, by the definition of signed interactions in the LSS model, it has two basic signed interactions, {(1,−1),(2,−1)} and {(1,+1),(2,+1)}. However, we can also write it as $1-1(X_{1}<0.5,X_{2}>0.5)-1(X_{1}>0.5,X_{2}<0.5)$, which has two different basic interactions, {(1,−1),(2,+1)} and {(1,+1),(2,−1)}. This means that a set of signed features that is an interaction in one of the representations is not an interaction in the other. Due to this identifiability problem, overlapping features can lead to both false positives and false negatives in terms of interaction recovery with RF. One may try to define interaction more broadly to avoid this identifiability problem. For the previous example $1(X_{1}<0.5,X_{2}<0.5)+1(X_{1}>0.5,X_{2}>0.5)$, although the basic signed interactions are not unique, they always constitute both *X*_1_ and *X*_2_. Whether the coefficients ${\left\{\beta_{j}\right\}}_{j=0}^{J}$ are allowed to have different signs also affects the identifiability. The previous example is identifiable if we only allow positive coefficients. For domain problems, where interactions are believed to be overlapping, one should investigate different identifiability conditions, but as this depends on the precise application, we leave this for future work. Our work in this paper provides a pathway to investigate this in detail later. We demonstrate how overlapping features affect our results with a simulation study in Section 4.

In Section 3, we show that a simple algorithm, LSSFind, that takes an RF tree ensemble as input can consistently recover basic interactions $S_{1},\ldots ,S_{J}$ in the LSS model. Besides recovering $S_{j}\subset [p]$, LSSFind can also recover the signs of each feature $k\in \cup_{j=1}^{J}S_{j}$ in the LSS model, which indicates whether the corresponding threshold behavior in Eq. **1** is given by a ≤- or ≥-inequality. Without loss of generality, in the rest of the paper, we assume that all inequalities are ≤ in Eq. **1**—that is,

[4] $$E(Y|X)=\beta_{0}+\sum_{j=1}^{J}\beta_{j}\prod_{k\in S_{j}}1(X_{k}\leq \gamma_{k}).$$

We stress, however, that all our results also hold for the general case Eq. **1**. Because we assume that all the features in basic interactions have minus signs, we denote $S_{1}^{-},\ldots ,S_{J}^{-}\subset [p]\times \{-1,+1\}$ with $S_{j}^{-}=\{(k,-1)\;:\;k\in S_{j}\}$ as basic signed interactions of the LSS model. As our theoretical results will show, the RF tree ensemble can recover not only the basic interactions $S_{j}\subset [p]$, but also basic signed interactions $S_{j}^{-}\subset [p]\times \{-1,+1\}$. In other words, through DWP and under the LSS model, the RF tree ensemble can recover not only which features interact with each other in the LSS model, but also whether a particular feature in an interaction has to be larger or smaller than some threshold for this interaction to be active. Besides basic signed interactions, we also define a “union signed interaction” as a union of individual basic signed interactions, as made more precise in the following definition.

Definition 1 (Union Signed Interactions): In the LSS model with basic signed interactions $S_{1}^{-},\ldots ,S_{J}^{-}\subset [p]\times \{-1,+1\}$, a (nonempty) set of signed features $S^{\pm }\subset [p]\times \{-1,+1\}$ is called a union signed interaction, if

[5] $$\begin{array}{l} S^{\pm }=\underset{j\in I}{\cup }S_{j}^{-}\;\underset{j\in I_{s},k\in S_{j},b_{k}\in \{-1,+1\}}{\cup }\{(k,b_{k})\}, \end{array}$$

for some (possibly empty) set of indices $I\subset \{j\in [J]\;:\;|S_{j}|>1\},\;I_{s}\subset \{j\in [J]\;:\;|S_{j}|=1\}$.

In other words, a union signed interaction is a union of one or more basic signed interactions. For a single-feature signed interaction, its sign-flipped counterpart can also be added to the union. For example, for an LSS model with $E(Y|X)=1(X_{1}\leq 0.5)+1(X_{2}<0.5,X_{3}<0.5),$ there are two basic signed interactions—namely, {(1,−1)} and {(2,−1),(3,−1)}—and five union signed interactions—namely, {(1,−1)}, {(2,−1),(3,−1)}, {(1,+1)}, {(1,−1),(2,−1),(3,−1)}, and {(1,+1),(2,−1),(3,−1)}.

The theoretical results that we present in Section 3 are asymptotic, in the sense that they assume the sample size *n* to go to infinity. Denote the number of signal features $\cup_{j=1}^{J}S_{j}$ in the LSS model to be *s*—i.e., $\sum_{j=1}^{J}|S_{j}|=s$. We assume *s* is uniformly bounded, regardless of *n* and *p*. However, the overall number of features *p* or the number of noisy features *p* – *s* can grow to infinity as *n* increases. Our theoretical results also assume

**C4** (Sparsity): $s=O(1)\;\text{and}\;\frac{\log\;(p)}{n}\to 0.$

This means that, in contrast to many theoretical works (16, 17, 40),^†^ our results hold in a high-dimensional setting, as long as the overall number of signal features *s* is bounded. The limit $\frac{\log\;(p)}{n}\to 0$ is a common assumption for high-dimensional settings when analyzing consistency properties of Lasso (see, for instance, refs. 41–43).

## 2. DWP for an RF Tree Ensemble

In this section, we first review the RF algorithm and then define DWP for a given RF tree ensemble.

### A. Review of RF.

RF is an ensemble of classification or regression trees, where each tree *T* defines a mapping from the feature space to the response. Trees are constructed on a bootstrapped or subsampled dataset $D^{\left(T\right)}$ of the original data D. Note that each tree is conditionally independent of one another, given the data. Any node *t* in a tree *T* represents a hyper-rectangle *R_t_* in the feature space. A split of the node *t* is a pair $\left(k_{t},\gamma_{t}\right)$, which divides the hyper-rectangle *R_t_* into two hyper-rectangles $R_{t,l}(k_{t},\gamma_{t})=R_{t}\cap 1(X_{k_{t}}\leq \gamma_{t})$ and $R_{t,r}(k_{t},\gamma_{t})=R_{t}\cap 1(X_{k_{t}}>\gamma_{t})$, corresponding to the left child *t_l_* and right child *t_r_* of node *t*, respectively. For a node *t* in a tree *T*, $N_{n}(t)=|\{i\in D^{\left(T\right)}:x_{i}\in R_{t}\}|$ denotes the number of samples falling into *R_t_*.

Each tree *T* is grown using a recursive procedure (denoted as the CART algorithm (2)), which proceeds in two steps for each node *t*. First, a subset $M_{\text{try}}\subset [p]$ of features is chosen uniformly at random. The size of $M_{\text{try}}$ is $m_{\text{try}}$. Then, the optimal split $k_{t}\in M_{\text{try}},\gamma_{t}\in R$ is determined by maximizing impurity decrease defined in Eq. **6**:

[6] $$\Delta_{I}^{n}(t)≔I_{n}(t)-\frac{N_{n}(t_{l})}{N_{n}(t)}I_{n}(t_{l})-\frac{N_{n}(t_{r})}{N_{n}(t)}I_{n}(t_{r}),$$

where *t_l_* (*t_r_*) is the left (right) child of *t*, and for sample size *n*, $I_{n}(t)$ is the impurity measure defined in this paper as

$$I_{n}(t)=\text{variance}\;\text{of}\{y_{i},i\in R_{t}\},$$

which is the variance of the response *y_i_*’s for all the samples in the region *R_t_*. Note that the analysis of this paper holds only for the variance impurity measure, but it is possible to extend to other impurities measures, which is left as future work. The procedure terminates at a node *t* if two children contain too few samples—e.g., $\min\{N_{n}(t_{l}),N_{n}(t_{r})\}\leq 1$—or if all responses are identical—e.g., $I_{n}(t)=0$. For any tree *T* and any leaf node $t_{\text{leaf}}\in T$, denote $p(t_{\text{leaf}})$ to be a path to that leaf node.

Definition 2 (Depth of a Path): Given a path $p(t_{\text{leaf}})$ that connects root node *t*_1_ and leaf node $t_{\text{leaf}}$ in a tree *T*, we define the depth of the path $p(t_{\text{leaf}})$ to be the number of nonroot nodes contained in the path.

For any hyper-rectangle *R_t_*, $\mu (R_{t})$ denotes its volume. We make the following assumptions on an RF tree ensemble:

Assumption 1 **(A1)** (Increasing Depth of a Tree in the RF Ensemble). *The minimum depth of any path in any tree goes to infinity—i.e.,*

$$\underset{T}{\min}\underset{t_{\text{leaf}}\in T}{\min}D(t_{\text{leaf}})\overset{p}{\to }\infty ,$$

*as* n→∞.

**A2** (Balanced Split in a Tree of the RF Ensemble). *Each split* $\left(k_{t},\gamma_{t}\right)$ *is balanced*: *for any node t*,

$$\min\left(\frac{\mu (R_{t,l}(k_{t},\gamma_{t}))}{\mu (R_{t,r}(k_{t},\gamma_{t}))},\frac{\mu (R_{t,r}(k_{t},\gamma_{t}))}{\mu (R_{t,l}(k_{t},\gamma_{t}))}\right)>\frac{C_{\gamma }}{1-C_{\gamma }}.$$

Note that, without loss of generality, we use the same $C_{\gamma }$ here as in the LSS model. Otherwise, we can always let $C_{\gamma }$ to be the minimum of the two.

**A3** ($m_{\text{try}}$ Is of Order p). $C_{m}p+(1-C_{m})s\leq m_{\text{try}}\leq (1-C_{m})(p-s)$, *where* $C_{m}\in (0,0.5)$ *is a constant.*

**A4** (No Bootstrap or Subsampling of Samples). *All the trees in RF are grown on the whole dataset without bootstrapping or subsampling*—*i.e.*, $D^{\left(T\right)}=D$ *for any T.*

A4 is a technical assumption that simplifies our notation and analysis. We assume that each tree is grown using all of the samples, which is quite different from the assumptions on subsampling in recent theoretical works on RF (e.g., refs. 15 and 17). The subsampling rate plays a crucial role in the analysis of the asymptotic distribution of the RF predictor (15, 17), where it is assumed that the subsampling rate converges to zero at a desirable rate. However, since we focus on the features selected at each node, and not on the asymptotic distribution of the predictor, we do not require such assumptions on the subsampling rate.

A1 ensures that the length of any decision path in any tree tends to infinity. This assumption is reasonable as tree depths in RF is usually of order O(log n), which tends to infinity as n→∞. A2 ensures that each node split is balanced. Similar conditions are used commonly in other papers (17). A3 shows the important role of the parameter $m_{\text{try}}$. Roughly speaking, $m_{\text{try}}$ cannot be too small or too big. When $m_{\text{try}}$ is too small, there will be too many splits on irrelevant features, which makes the tree noisy. When $m_{\text{try}}$ is too big, there will be too little variability in the tree ensemble. This motivation will be made rigorous in the proof of Theorem 2.

### B. DWP.

In this section, for a tree ensemble from RF, we formally introduce DWP. Given a decision tree *T* in an RF tree ensemble, we can randomly select a path P of *T* as follows: We start at the root node of *T* and then, at every node, randomly go left or right until we reach a leaf node. This is equivalent to selecting a path in *T* of depth *D* with probability $2^{-D}$ from all the paths in a decision tree. Denote the nodes in P to be $t_{1},\ldots ,t_{D},t_{\text{leaf}}$. As such, any path P in a decision tree *T* can be associated with a sequence of signed features $\left(k_{t_{1}},b_{t_{1}}),\ldots ,(k_{t_{D}},b_{t_{D}})\in [p]\times \{-1,+1\right\}$, where *D* is the depth of the path, and for any inner node t∈[D] on the path, the sign *b_t_* indicates whether the path at node *t* followed the ≤ direction ($b_{t}=-1$) or the direction ($b_{t}=+1$) for the split on feature $k_{t}\in [p]$. For a given RF tree ensemble depending on data D, the randomly selected path P of tree *T*, and any fixed constant ϵ>0, we now define ${\hat{F}}_{\epsilon }(P,T,D)$ to be the set of signed features on P, where the corresponding node in the RF had an impurity decrease of at least *ϵ*—that is,

[7] $$\begin{array}{ll} & {\hat{F}}_{\epsilon }(P,T,D)≔\{(k_{t},b_{t})\;|\;t\;\text{is}\;\text{an}\;\text{inner}\;\text{node}\;\text{of}\;P\\ & \text{with}\;\;\Delta_{I}^{n}(t)>\epsilon \;\text{and}\;\text{feature}\;k_{t}\;\text{appears}\;\text{first}\;\text{time}\;\text{on}\;P\}. \end{array}$$

We use ${\hat{F}}_{\epsilon }$ as a shorthand for ${\hat{F}}_{\epsilon }(P,T,D)$ when the path P from tree *T* and the data D of interest are clear. Note that if a feature appears more than once on the path P, its sign in ${\hat{F}}_{\epsilon }$ is the sign when the feature appears the first time with the impurity decrease above the threshold. Our main theorem will be stated in terms of the DWP of a signed feature set $S^{\pm }\subset [p]\times \{-1,+1\}$ on the random path P within ${\hat{F}}_{\epsilon }$. To formally define the DWP of $S^{\pm }$, we first need to identify the sources of randomness underlying ${\hat{F}}_{\epsilon }$. There are three layers of randomness involved:

1. **(**D **: Data randomness)**: The randomness involved in the data generation;
2. (***T*: Tree randomness**): The randomness involved in growing an individual tree with parameter $m_{\text{try}}$, given data D;
3. **(**P **: Path randomness)**: The randomness involved in selecting a random path P of depth *d* with probability $2^{-d}$, given a tree *T* from an RF tree ensemble with parameter $m_{\text{try}}$ based on data D.

In the following definition of the DWP of signed feature sets, the probability is conditioned on data D and taken only over the randomness of the tree *T* and the randomness of selecting one of its paths, as in P.

Definition 3 (DWP): Conditioning on data, for any signed feature set $S^{\pm }\subset [p]\times \{-1,+1\}$, we define the DWP of $S^{\pm }$ as the probability that $S^{\pm }$ appears on the random path P within the set ${\hat{F}}_{\epsilon },$—that is,

[8] $${\text{DWP}}_{\epsilon }(S^{\pm })=P_{\left(P,T\right)}(S^{\pm }\subset {\hat{F}}_{\epsilon }\;|\;D).$$

We emphasize that the probability of selecting a path in a tree T is $P(P|T)=2^{-d}$, where d is the depth of the path P.

While we only have a fixed sample size, which means that the data randomness is inevitable, the tree randomness and path randomness are generated by the algorithm and thus can be eliminated by sampling as many trees and paths as we like. Because the DWP in Eq. **8** is only conditioned on data, for any given ϵ>0 and set of signed features $S^{\pm }$, it can be computed with arbitrary precision from an RF tree ensemble with sufficiently many trees (recall that, conditioned on data D, the different trees in an RF tree ensemble are generated independently).

## 3. Main Results

In this section, we present our main theoretical results, which are concerned with DWP, as introduced in the previous section. Our results show that LSSFind (Algorithm 1), which is based on DWP at an appropriate level *ϵ* described in Theorem 3, consistently recovers signed interactions under an LSS model. Before we state our main results in full detail, we want to illustrate it with a simple example.

Algorithm 1: LSSFind ($m_{\text{try}}$,*ϵ*, *η*, $s_{\max}$)

**Input:** Dataset D, RF hyperparameter *m_try_*, impurity threshold ϵ>0, prevalence threshold η>0, and maximum interaction size $s_{\max}\in N$.

**Output:** A collection of sets of signed features. Train an RF using dataset D with parameter *m_try_*; return $\left\{S^{\pm }\subset [p]\times \{-1,+1\right\}$ such that $|S^{\pm }|\leq s_{\max}$ and $2^{\left|S^{\pm }\right|}\cdot {\text{DWP}}_{\epsilon }(S^{\pm })\geq 1-\eta \}.$

**Illustrative Example:** Assume that p=2, and there are just two features *X*_1_ and *X*_2_. Assume there is a single interaction J=1, and the regression function is given by

[9] $$E(Y|X_{1},X_{2})=1(X_{1}\leq 0.5)\cdot 1(X_{2}\leq 0.5).$$

**Fig. 1.**
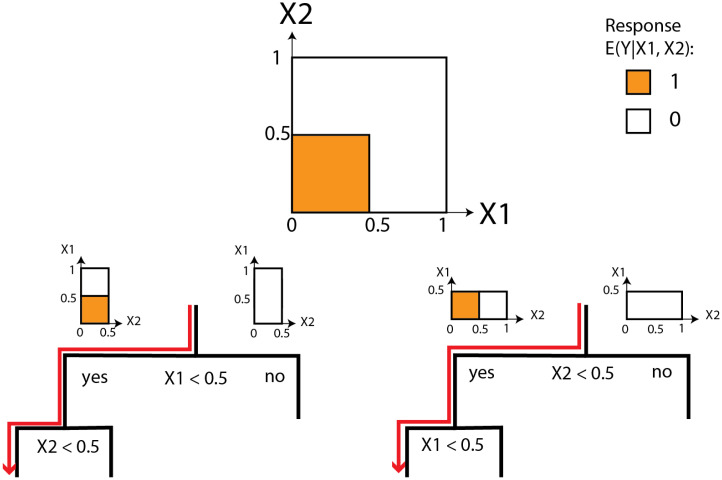
Exemplary RF decision trees trained on data as in Eq. **9** to illustrate the results that will appear in Theorem 2. (*Upper*) Response surface of $E\left(Y|\;X_{1},X_{2}\right)$, as in Eq. **2**, with $X_{1}\in [0,1]$ on the *x* axis and $X_{2}\in [0,1]$ on the *y* axis. (*Lower Left*) A decision tree that splits on feature *X*_1_ at the root node with the respective regions and conditional response surfaces for the left and right child of the root node. (*Lower Right*) A decision tree that splits on feature *X*_2_ at the root node. The red-marked decision paths contain all signed features from the basic signed interaction $S^{-}=\{(1,-),(2,-)\}$ from an LSS model, as in Eq. **9**. For both of the trees, if one starts at the root node and randomly goes left or right at every node, then the probability of the basic signed interaction to appear on the path is ${\text{DWP}}_{\epsilon }(S^{-})=2^{-2}=2^{-|S^{-}|}$. In contrast, for any other set of signed features $S^{\pm }\subset [p]\times \{-1,+1\}$, it holds that ${\text{DWP}}_{\epsilon }(S^{\pm })<2^{-|S^{\pm }|}$. This provides a simple example for the more general result in Theorem 2.

The response surface of Eq. **9** is shown in Fig. 1, *Upper*. We consider the population case, where we have full access to the joint distribution *P*(*X*,  *Y*)—that is, we have access to an unlimited amount of data (n=∞). When we apply the RF algorithm as in Section 2, then, for each individual tree in the forest, the root node either splits on feature *X*_1_ or on feature *X*_2_. Since *X*_1_ and *X*_2_ are completely symmetric in the distribution *P*(*X*,  *Y*), thus, if the RF algorithm grows more and more trees, in the limit, half of them will split on *X*_1_ at the root node and half of them split on *X*_2_ at the root node. For infinite data, this 50/50 split is introduced by the CART algorithm, since the two splits have identical decreases of impurities. Furthermore, the split at any node will be at 0.5 for any of the two features, since the two splits corresponding to $X_{1}\leq 0.5$ and $X_{2}\leq 0.5$ maximize the impurity decrease given infinite data. This is illustrated in Fig. 1, where *Left Lower* shows a tree that splits on feature *X*_1_ at the root node, and *Right Lower* shows a tree that splits on feature *X*_2_ at the root node. As each tree in RF grows to purity, when the root node splits at feature *X*_1_, then, for the path of the tree that follows the (1,+1) direction—that is, the $X_{1}>0.5$ direction—the tree will stop growing, as the respective response surface is already constant. However, for the path of the tree that follows the (1,−1) direction—that is, the $X_{1}\leq 0.5$ direction—the tree will further split on the remaining feature *X*_2_. Then, the tree will stop because the node reaches purity. Thus, we conclude that the forest consists of exactly the two different trees shown in Fig. 1 and in the limit, where the number of trees grows to infinity, each of the two trees appears equally often.

For each node *t* in these trees, the impurity decrease satisfies $\Delta_{I}^{n}(t)\geq 1/16$. Thus, for any ϵ<1/16, we can show that the DWP of the basic signed interaction $S^{-}=\{(1,-1),(2,-1)\}$ is $2^{-|S^{-}|}$. To show this, we can get:

$$\begin{array}{ll} & {\text{DWP}}_{\epsilon }(S^{-})=P(S^{-}\subset {\hat{F}}_{\epsilon }|D)\\ & =\underset{=0.5,\;\;\text{correspond}\;\text{to}\;\text{the}\;\text{left}\;\text{tree}}{\underset{︸}{P_{T}(T'\text{s}\;\text{root}\;\text{splits}\;\text{on}\;\text{feature}1)}}\\ & \cdot \underset{=0.25,\;\text{only}\;\text{the}\;\text{red}\;\text{path}\;\text{satisfies}\;\text{this}.}{\underset{︸}{P(S^{-}\subset {\hat{F}}_{\epsilon }|D,T'\text{s}\;\text{root}\;\text{splits}\;\text{on}\;\text{feature}1)}}+\\ & \underset{=0.5,\;\text{correspond}\;\text{to}\;\text{the}\;\text{right}\;\text{tree}}{\underset{︸}{P_{T}(T'\text{s}\;\text{root}\;\text{splits}\;\text{on}\;\text{feature}2)}}\\ & \cdot \underset{=0.25,\;\text{only}\;\text{the}\;\text{red}\;\text{path}\;\text{satisfies}\;\text{this}.}{\underset{︸}{P(S^{-}\subset {\hat{F}}_{\epsilon }|D,T'\text{s}\;\text{root}\;\text{splits}\;\text{on}\;\text{feature}2)}}\\ & =0.5\cdot 2^{-2}+0.5\cdot 2^{-2}=2^{-2}=2^{-|S^{-}|}. \end{array}$$

In the above example with infinite data, the tree depth is not going to infinity, which means it does not satisfy A1. A1 is needed only for the finite sample case because, for finite samples, internal nodes in a tree can never reach purity due to noise.

In Fig. 1, the paths that contain the basic signed interaction $S^{-}=\{(1,-1),(2,-1)\}$ are marked red. For all the other sets of signed features $S^{\pm }\subset [p]\times \{-1,+1\}$, it is easy to check that ${\text{DWP}}_{\epsilon }(S^{\pm })<2^{-|S^{\pm }|}.$ For example,

$${\text{DWP}}_{\epsilon }(\{(1,-1),(2,+1)\})=0.5\cdot 2^{-2}+0.5\cdot 0<2^{-2},$$

and

$$\begin{array}{ll} & {\text{DWP}}_{\epsilon }(\{(1,-1)\})\\ & =\underset{=0.5,\;\text{correspond}\;\text{to}\;\text{the}\;\text{left}\;\text{tree}}{\underset{︸}{P_{T}(T'\text{s}\;\text{root}\;\text{splits}\;\text{on}\;\text{feature}1)}}\\ & \cdot \underset{=0.5,\;\;\text{any}\;\text{path}\;\text{that}\;\text{goes}\;\text{left}\;\text{at}\;\text{the}\;\text{root}\;\text{satisfies}\;\text{this}.}{\underset{︸}{P(\{(1,-1)\}\subset {\hat{F}}_{\epsilon }|D,T'\text{s}\;\text{root}\;\text{splits}\;\text{on}\;\text{feature}1)}}+\\ & \underset{=0.5,\;\text{correspond}\;\text{to}\;\text{the}\;\text{right}\;\text{tree}}{\underset{︸}{P_{T}(T'\text{s}\;\text{root}\;\text{splits}\;\text{on}\;\text{feature}2)}}\\ & \cdot \underset{=0.25,\;\text{only}\;\text{the}\;\text{red}\;\text{path}\;\text{satisfies}\;\text{this}.}{\underset{︸}{P(S^{-}\subset {\hat{F}}_{\epsilon }|D,T'\text{s}\;\text{root}\;\text{splits}\;\text{on}\;\text{feature}2)}}\\ & =0.5\cdot 2^{-1}+0.5\cdot 2^{-2}<2^{-1}. \end{array}$$

As we will formally state in the two theorems below, the same reasoning holds true asymptotically for any RF trained on the data from the LSS model—namely, the DWP of a set of signed features $S^{\pm }\subset [p]\times \{-1,+1\}$ is always upper-bounded by $2^{-|S^{\pm }|}$, and this upper bound is attained if and only if $S^{\pm }$ is a union-signed interaction. Recall that the DWP depends on the data D. It turns out that the general upper bound follows directly from the construction of DWP and holds for any data D—i.e., independent of the LSS model—as the following theorem shows.

Theorem 1. *For any impurity threshold* ϵ>0 *and any set of signed features* $S^{\pm }\subset [p]\times \{-1,+1\}$ *for the RF algorithm from Section 2, it holds true that*

• (*General upper bound*) ${\text{DWP}}_{\epsilon }(S^{\pm })\leq 2^{-|S^{\pm }|}.$

In addition, when the data D are generated from an LSS model, asymptotically (as the sample size increases), the general upper bound is attained if and only if $S^{\pm }$ is a union signed interaction, as the following theorem shows.

Theorem 2. *Assume that the data* D *are generated from an LSS model with uniformity, bounded-response, nonoverlap basic interactions, and sparsity constraints* (*see C*1–*C*4). *For any impurity threshold* ϵ>0, *let*

[10] $$b(\epsilon )≔{\left(4\epsilon /(C_{\beta }^{2}C_{\gamma }^{2s-1})\right)}^{C_{m}^{2s}/\log\;(1/C_{\gamma })},$$

*with constants* $C_{\beta }$ *as in* *Eq.* **2**, $C_{\gamma }$ *as in* *Eq.* **3**, *s as in C*4, *and C_m_ as in A*3. *Given a set of signed features* $S^{\pm }\subset [p]\times \{-1,+1\}$, *for the RF algorithm from Section 2, it holds true that*,

• (*Interaction lower bound*) *when* $S^{\pm }$ *is a union signed interaction as in* *Definition* 1, *we have*
  $${\text{DWP}}_{\epsilon }(S^{\pm })\geq 2^{-|S^{\pm }|}-b(\epsilon )-r_{n}(D,\epsilon );$$
• (*Noninteraction upper bound*) *when* $S^{\pm }$ *is not a union signed interaction, then*,
  $${\text{DWP}}_{\epsilon }(S^{\pm })\leq 2^{-|S^{\pm }|}\left(1-\frac{C_{m}^{s}}{2}\right)+r_{n}(D,\epsilon ),$$

*with*

$$r_{n}(D,\epsilon )\overset{p}{\to }0\;\text{as}n\to \infty ,$$

*where* $\overset{p}{\to }$ *denotes convergence in probability*.

Proof Sketch: The detailed proof of Theorem 2 is deferred to *SI Appendix*, Section S1. It has two major parts: first, showing the assertion for the idealized population case and, second, extending the population case to the finite sample case.

In the first part, we define a population version of the set ${\hat{F}}_{\epsilon }$, which we denote as F. The set F only contains desirable features, which are features of a path P that correspond to a positive decrease in impurity if the RF gets to see the full distribution P(X,Y) (not just a finite sample D). Note that desirable/nondesirable features are different from signal/noisy features. The definition of desirable/nondesirable features depends on the concerned path in a tree. A noisy feature is always a nondesirable feature, but a signal feature can become a nondesirable feature when it has been split in the path. See *SI Appendix*, Definition S1. The set F is an oracle, in the sense that its construction depends on the true underlying LSS model. This is in contrast to the set ${\hat{F}}_{\epsilon }$, which can be computed for any given path from a tree of RF. Given this definition of F, a sketch of the proof of the major assertions of Theorem 1 and 2 is as follows:

1. When a set of signed features $S^{\pm }$ appears in F, this implies that every time a signed feature $(k,b)\in S^{\pm }$ appears on the way from the root node to the leaf, the splitting direction implied by *b* was selected for P, which gives rise to the general upper bound of ${\text{DWP}}_{\epsilon }(S^{\pm })\leq 2^{-|S^{\pm }|}$ (Theorem 1).
2. If $S^{\pm }$ is a union interaction, then (assuming all leaf nodes of the tree are pure) a correct splitting direction for each of its features already implies that $S^{\pm }$ appears on P and, thus, ${\text{DWP}}_{\epsilon }(S^{\pm })\approx 2^{-|S^{\pm }|}$ (see first part of Theorem 3).
3. If $S^{\pm }$ is not a union interaction, then there will always be the possibility that, although every split for an encountered feature that is an element of $S^{\pm }$ was done in the correct direction, some of the features in $S^{\pm }$ were just never encountered, and, therefore, a correct splitting direction does not imply that $S^{\pm }$ appears on P; hence, ${\text{DWP}}_{\epsilon }(S^{\pm })<2^{-|S^{\pm }|}$ (see second part of Theorem 3).

In the second part of the proof, we show that the observed set ${\hat{F}}_{\epsilon }$ and the oracle set F are the same, with probability going to one as *ϵ* goes to zero and *n* goes to infinity. That would be nice and easy if a tree grown using finite samples will converge to a tree grown using the population in terms of the splitting features and thresholds when sample size tends to infinity. However, that is not true. The obstacle is that, when a node splits on a nondesirable feature, since all the thresholds yield the same impurity decrease in the population case, the threshold selected via finite samples can deviate from the threshold via the population, no matter how many samples are used. Thus, we need to carefully analyze desirable features and nondesirable features separately based on uniform convergence results. □

Remark 1 : Theorems 1 and 2 demonstrate that recovery of interactions becomes exponentially more difficult as the size of an interaction increases. An interaction $S^{\pm }$ corresponds to a region of size $O(2^{-|S^{\pm }|})$, which means the sample size must be much larger than $2^{\left|S^{\pm }\right|}$ to have enough samples in that region. Also, the DWP at an appropriate level *ϵ* of a basic interaction $S^{\pm }$ is $2^{-|S^{\pm }|}$. To have a consistent estimate, the number of independent paths should be much larger than $2^{\left|S^{\pm }\right|}$. Thus, when one wants to recover an interaction of size *s*, the number of samples and the number of trees must be much larger than $2^{s}$. That shows the intrinsic difficulty of estimating high-order interactions.

Using the conclusions in Theorem 2, one can show that LSSFind (Algorithm 1) can consistently recover all the basic interactions from the LSS model, as stated in Theorem 3.

Theorem 3. *Let the output of LSSFind* (*Algorithm* 1) *be* $S(m_{\text{try}},\epsilon ,\eta ,s_{\max})$. *Under the same settings as in* *Theorem* 2, *as long as* $m_{\text{try}},\epsilon ,\eta$ *satisfies the assumptions in* *Theorem* 2 *and the following condition*:

[11] $$2^{s}\cdot b(\epsilon )<\eta <\frac{{\left[C_{m}\right]}^{s}}{2},$$

*with* b(ϵ) *defined in* *Eq.* **10** *and C_m_ in A*3, *then, with probability approaching one as* n→∞, S *is a superset of the basic signed interactions with size at most* $s_{\max}$ *and a subset of union signed interactions. In particular, if we define*

U={S∈S|There is no set  S′∈S  s.t.,  S⊊S′},

*then* U *equals the set of basic signed interactions of size at most* $s_{\max}$.

Note that to recover all the basic interactions, $s_{\max}$ needs to be larger than or equal to the order of all the basic signed interactions, but the latter is unknown, as we do not know the underlying LSS model.

Proof : If $S^{\pm }$ is not a union signed interaction, then it follows from the second part of Theorem 2 and $\eta <{\left[C_{m}\right]}^{s}/2$ that $2^{\left|S^{\pm }\right|}\cdot {\text{DWP}}_{\epsilon }(S^{\pm })<1-\eta ,$ with probability approaching one as n→∞. Thus, S is a subset of union signed interactions. If $S^{\pm }$ is a basic signed interaction of size at most $s_{\max}$, then it follows from the first part of Theorem 2 and the fact that $2^{s}\cdot b(\epsilon )<\eta$ that $2^{\left|S^{\pm }\right|}\cdot {\text{DWP}}_{\epsilon }(S^{\pm })\geq 1-\eta ,$ with probability approaching one as n→∞. Thus, S is a superset of the basic signed interactions with size at most $s_{\max}$. □

Remark 2 : One important assumption in our theorem is the sparsity of signal features. If there are many “weak” signal features, it is very hard for RF to work well. For RF, at each node of a tree, only one feature is used. That means the total number of features used along each path is limited by the depth of the tree, which is usually of order O(log n). For our assertions of Theorem 2, the hard threshold *ϵ* in the set ${\hat{F}}_{\epsilon }$ has the purpose to select the signal features. Clearly, the choice of an appropriate value of *ϵ* is hard in practice. The fitting procedure in iRFs (11) (which uses joint prevalence on decision paths in RF to recover interactions, similar as suggested by Theorem 2) filters noisy features not with a hard, but with a soft thresholding procedure: It grows several RFs iteratively and samples features at each node, according to their feature importance from the previous iteration. In that way, one does not need to chose a single hard threshold, which leads to a much more practical algorithm. Unfortunately, such an iterative soft thresholding makes theoretical analysis much harder, which is why we restrict to the hard threshold for the theoretical analysis in this work.

One of the remarkable aspects of the result in Theorem 3 is that the range of *η* is independent of any model coefficients in the LSS model (that is, the linear *β* coefficients and the *γ* thresholds). For sufficiently small *ϵ*, it only depends on the number of signal features *s* and the bound of $m_{\text{try}}$—i.e., *C_m_*—and nothing else. In a sense, this shows that the tree ensemble of RF contains the qualitative or discrete-set information of which features interact with each other, independently of the quantitative information about what are the numerical parameters or model coefficients in the LSS model.

Another interesting aspect about the results from Theorem 3 is that they shed some light on the influence of *m_try_* on the interaction recovery performance of RF. For the third assertion in Theorem 2, we actually show that

$$\begin{array}{l} {\text{DWP}}_{\epsilon }(S^{\pm })\leq r_{n}(D,\epsilon )\;+\\ 0.5^{\left|S^{\pm }\right|}\left(1-0.5\underset{k\in \cup_{j}S_{j}}{\min}P(\text{root}\;\text{node}\;\text{splits}\;\text{on}\;\text{feature}\;k)\right). \end{array}$$

When *m_try_* is too large, $\min_{k\in \cup_{j}S_{j}}P(\text{root}\;\text{node}\;\text{splits}\;\text{onfeature}\;k)$ can get very small, as particularly strong features (large initial impurity decrease) can mask weaker features. As an extreme example, consider the situation where $m_{\mathrm{try}}=p$, and, thus, the root node gets to see all the features. In that case, the single feature that has the highest impurity decrease, say, *X*_1_, will always appear at the root node, and, hence, for $S^{\pm }=\{(1,-1)\}$ or $S^{\pm }=\{(1,+1)\}$, one will get ${\text{DWP}}_{\epsilon }(S^{\pm })=2^{-|S^{\pm }|}=0.5$, independent of whether $S^{\pm }$ is an interaction or not. This shows that when *m_try_* is too large, DWPs corresponding to false interactions can attain the universal upper bound $2^{-|S^{\pm }|}$, which leads to false positives in terms of interaction recovery. On the other hand, when *m_try_* is too small, for a signal feature $k\in \cup_{j}S_{j}$, it can take a long time until it gets selected into the candidate feature set at a node. In particular, for a finite sample, it can happen that the tree reaches purity due to lack of samples without having split on any of the signal features. Hence, the reasoning of Theorem 2—namely, that correct split direction + pure path implies that a union interaction appears on the path does not hold anymore. This can lead to union interactions having significantly smaller DWP than the universal upper bound $2^{-|S^{\pm }|}$—i.e., false negatives in terms of interaction recovery.

## 4. LSSFind and Simulation Results

In this section, motivated by our theoretical results in the previous section, we evaluate LSSFind empirically in terms of its ability to recover interactions.^‡^ Simulated experiments are carried out to assess the ability of LSSFind to correctly recover interactions from the LSS model, even when some of the LSS model assumptions are violated.

In LSSFind, one needs to search over all possible sets with size at most $s_{\text{max}}$ to obtain the final result. That is computationally very intensive. One more efficient way is to only look for sets with size at most $s_{\text{max}}$ and also with

[12] $${\text{DWP}}_{\epsilon }(S^{\pm })\geq (1-\eta )\cdot 2^{-s_{\text{max}}},$$

which implies that we don’t need to search over all possible sets with sizes at most $s_{\text{max}}$; instead, we need to search only for sets whose $DWP_{\epsilon }$’s are larger than $(1-\eta )\cdot 2^{-s_{\text{max}}}$. Because many sets with sizes at most $s_{\text{max}}$ are filtered out, this significantly reduces the search space. We use the FP-growth algorithm (44) to obtain those sets of signed features that have a DWP higher than some threshold. Note that DWP requires an infinite number of trees. To approximate DWP, we use 100 trees in the simulation. Since each tree contains thousands of paths, we have hundreds of thousands of paths to estimate the DWP for.

### A. Simulated Data from LSS Models.

In the following, we present simulation results, where we generated data D from the LSS model for different numbers and orders of basic interactions and different signal-to-noise ratios (SNRs). We find that LSSFind recovers the true interactions from the LSS model with high probability whenever the overall number of basic interactions and their orders are small.

More precisely, we consider *p* = 20 features and *n* = 1, 000 samples, where each feature *X_j_* is generated from an uniform distribution U([0,1]), independent from one another. The number of basic interactions is denoted as *J*, and the order of each interaction is denoted by *L*. We consider the same threshold *τ* for all features. The noise is Gaussian with variance $\sigma^{2}$, and the response is:

[13] $$Y=\sum_{j=1}^{J}\prod_{k=(j-1)\cdot L+1}^{k\cdot L}1(X_{k}<\tau )+N(0,\sigma^{2}).$$

We consider different values for *J*, *L*, and $\sigma^{2}$—namely, *J* = 1, 2, *L* = 2, 3, 4, and $\sigma^{2}$ s such that the SNR is 0.5, 1, 2, or 5. For a given *J* and *L*, the threshold *τ* is chosen such that about 50% of samples fall into the union of hyper-rectangles—that is, $\cup_{j=1}^{J}\cap_{k=(j-1)\cdot L+1}^{j\cdot L}\{X_{k}<\tau \}$. As we know that the number of samples falling into $\cup_{j=1}^{J}\cap_{k=(j-1)\cdot L+1}^{j\cdot L}\{X_{k}<\tau \}$, which can also be roughly thought as the label imbalance, has a high impact on the results, keeping this number the same across different simulation settings makes sure that the simulation outcomes are more comparable. The results are averaged across 40 independent Monte Carlo runs. We grow RF using the scikit-learn package with 100 trees. We apply LSSFind with parameters η=0.01, ϵ=0.01, and $s_{\max}=L+1$. Recall that we use Eq. **12** to select candidate interactions. If $s_{\max}$ is set to *L*, the condition Eq. **12** would be too restrictive for challenging situations, such as when the LSS model is violated, and LSSFind can end up finding no interactions. Given a set $S^{*}$ of *K* true basic signed interactions from the respective LSS model and output from LSSFind S, we evaluate their proximity based on their Jaccard distance:

[14] $$\text{score}(S^{*},S)=\frac{\left|S^{*}\cap S\right|}{\left|S^{*}\cup S\right|}.$$

Note that any element in $S^{*}$ and S is a set of signed features. This score gives no credit for partial recovery: If one interaction $S^{\pm }$ in $S^{*}$ is {(1,+1),(2,+1)}, there will be no credit for S if it contains subsets of $S^{\pm }$, such as {(1,+1)}, or same features with different signs, such as {(1,+1),(2,−1)}. While this score can be overly restrictive for practical problems, it is suitable for our simulation because we would like to evaluate whether LSSFind can consistently recover the interactions in the LSS model. The simulation results are shown in *SI Appendix*, Fig. S1. In general, the performance of LSSFind sharply degrades when the number of basic interactions and the order of interactions increases. For *K* = 1 and *L* = 2, 3, 4, LSSFind almost always recovers the correct basic signed interactions. For K=L=2, it mostly recovers the correct basic signed interactions, except for small SNR. When *K* = 2 and *L* = 3, 4, LSSFind rarely recover the basic signed interactions for this simulation setup, resulting in a score of almost zero. Note that this is consistent with our results in Theorem 2, which indicates that the problem is much harder for more interactions and higher-order interactions. We also explored the high-dimensional case. When p=20,50,100,200 and n=1000·(1+log (p/20), the score for LSSFind is shown in *SI Appendix*, Fig. S5. The scaling of *p* and *n* is chosen to make sure log p/n≈0.001, and also when *p* = 20, *n* will be 1,000, which corresponds to our previous numerical setting for better comparison. Recall that Theorems 2 and 3 require condition log (p)/n→0, as stated in condition C4. We also note that log (p)/n→0 is commonly imposed when analyzing lasso problems, too (41–43). As can be seen in *SI Appendix*, Fig. S5, the score increases and approaches to one as the dimension *p* increases. This is consistent with Theorem 3, which shows that LSSFind can recover the underlying interactions for the high-dimensional case.

### B. Robustness to LSS Model Violations.

In the following, we present simulation results for LSSFind when the data are generated from a misspecified LSS model, which means that some of the LSS model assumptions are violated. We find that LSSFind deteriorate when the LSS model is violated. We consider a misspecified LSS model with *SNR* = 5 and two order-2 interactions with *p* = 20 features and *n* = 1, 000 samples, analog as in *SI Appendix*, Fig. S1, second row, first column, third bar. We consider the following violations of LSS model assumptions:

• **Overlapping interactions:** Different basic interactions have overlapping features. When overlap=1, the basic interactions are ((1,−1),(2,−1)), ((2,−1), (3,−1)).
• **Correlated features:** Different features are correlated instead of independent. When corr=α, the correlation between feature *j*_1_ and *j*_2_ is $\alpha^{\left|j_{1}-j_{2}\right|}$.
• **Heavy-tail noise:** Tthe noise follows a Laplace or Cauchy distribution, which have heavier tails than (sub)Gaussian distributions. The noise is normalized such that the SNR is 50.

Results of LSSFind are shown in *SI Appendix*, Fig. S2. For heavy-tail noise, we observe a gradual drop in performance. For the correlated feature case, one can see that LSSFind has reasonable performance when the correlation is close to zero, but its performance deteriorates when the correlation is high. Similarly, for the overlapping feature case, the performance worsens.

### C. Empirical Comparison between LSSFind and iRF.

Our original motivation to study DWP in RF tree ensemble came from the strong positive empirical evidence of iRF (11, 13). There are three major reasons why the full iRF procedure is hard to analyze theoretically: First, the iterative reweighting in iRF is based on the feature importance metric of MDI. Analyzing MDI for the RF algorithm is a challenging task on its own. In particular, MDI of noisy features in deep trees are known to have a bias (6–8), which may propagate through various iterations in iRF and make a theoretical analysis very challenging. Second, the iRF procedure selects interactions from the paths of the RF tree ensemble via the RIT algorithm (12). Thereby, individual paths are weighted according to the number of observations which fall into their respective leaf nodes. This means that the selected feature interactions of iRF cannot be derived from the RF tree ensemble directly, but depend on the data in a more complex way. Third, the outer stability layer of iRF, where interactions are evaluated based on their consistent appearance among several bootstrap replications of the procedure, adds an additional layer of complexity for theoretical analysis.

In order to still analyze the major aspects of iRF theoretically, we proposed the related LSSFind algorithm. Instead of iterative reweighting via MDI, LSSFind introduces a single hard threshold on the impurity index at individual tree nodes. Moreover, instead of selecting interactions via RIT, LSSFind is based on DWP, which is derived from the tree ensemble directly, without an additional data-dependent sampling scheme. In other words, although a high DWP in LSSFind does not exactly correspond to the RIT interaction selection strategy employed in iRF, they both build on similar high-level quantities—namely, sets of stable features, which often appear together on decision paths in an RF tree ensemble. Therefore, our theoretical results on DWP and LSSFind provide evidence that the general interaction discovery strategy of iRF is theoretically justified. In the following, we complement our theoretical findings about LSSFind with an empirical comparison between iRF and LSSFind.

We consider the same simulation setting as in Section A. However, we replace the very strict performance measure in Eq. **14** by a weaker one.^§^ Specifically, given a set $S^{*}$ of *K* true basic signed interactions from the respective LSS model and output from LSSFind and iRF, respectively, S, we now evaluate their proximity based on:

[15] $$\bar{\text{score}}(S^{*},S)=\frac{\left|\{\cup_{S^{-}\in S^{*}}S\}\cap \{\cup_{S^{-}\in S}S\}\right|}{\left|\{\cup_{S^{-}\in S^{*}}S\}\cup \{\cup_{S^{-}\in S}S\}\right|}.$$

Note that Eq. **15** corresponds to the Jaccard distance on the set of unsigned features that appear in any of the detected interactions. While the stricter metric in Eq. **14** is more appropriate to evaluate finite sample validity of Theorem 2, the relaxed version in Eq. **15** is arguably of more practical interest. This is because it gives partial credit for interactions that are almost, but not perfectly, recovered. If $\bar{\text{score}}(S^{*},S)$ is high, it means that the features in the discovered interactions overlap with the features in the true interactions, which would greatly narrow down the interaction search space and save tremendous effort for subsequent analysis for a practical problem.

For the iRF algorithm, we used the signed iRF algorithm (siRF) from the Python iRF package iRF,^¶^ with default parameter settings and a threshold on iRF’s stability score of 0.5 for interaction selection, as recommended in ref. 13. Simulation results are shown in *SI Appendix*, Fig. S3. When the LSS model as in Eq. **13** is relatively simple—for example, when it has only a single signed interaction (*K* = 1) or only a single feature per signed interaction (*L* =1)—iRF and LSSFind perform comparably (first row and second row, first column, of *SI Appendix*, Fig. S3). However, when the LSS model gets more complex, with several additive interactions (*K* > 1) each having more than one signed features (L>1) (second row, second and third columns in *SI Appendix*, Fig. S3), iRF outperforms LSSFind in terms of the metric Eq. **15**.^ǁ^ In summary, we find that iRF outperforms LSSFind in situations where the underlying LSS model is more complex and when a flexible performance metric is chosen. This appears to be consistent with the fact that the iRF algorithm has witnessed empirical success on specific domain data problems (11), whereas LSSFind was specifically constructed in such a way that it reflects our result in Theorem 2.

## 5. Discussion

Relevant statistics theory starts with a model that is a good approximation to reality. Thus, it is important to derive theoretical results under a model that is scientifically motivated. Our proposed LSS model class provides such a family that reflects the biological phenomena of biomolecules interacting through thresholding. Also, analyzing RF-based algorithms under different models, rather than the smoothness classes in the literature, can give insights into their empirical adaptivity. Our results give a theoretical result that DWP of a set of features in an RF tree ensemble recovers high-order interactions under the LSS model and reasonable conditions on the RF hyperparameters. Moreover, the universality of interaction’s DWP in LSS models gives insights into the general difference between quantitative (e.g., prediction accuracy) and qualitative (e.g., interaction recovery) information extraction. In scientific problems, often the latter is of higher interest. Thus, this work narrows the gap between theory and practice for Boolean interaction discovery and is of general interest to the fields of statistics, data science, ML, and scientific fields, such as genomics.

Our theoretical analysis also gives some insights of RF for tuning a crucial hyperparameter $m_{\text{try}}$: Given an interaction with a fixed size, the noninteraction DWP upper bound in Theorem 2 depends only on *C_m_*, and *C_m_* is only constrained by $m_{\text{try}}$ (A3). Therefore, one can find an optimal $m_{\text{try}}$ that minimizes this upper bound. The optimal choice of $m_{\text{try}}$ turns out to be $m_{\text{try}}^{⋆}=p\cdot (0.5-s/(2(p-2))$. If one-third of all features are signal features—that is, s=p/3 —$m_{\text{try}}^{⋆}$ recovers the default choice in standard RF implementations for regression—namely, $m_{\text{try}}^{⋆}\approx p/3$. However, when p≫s, the optimal choice from our theoretical results corresponds to $m_{\text{try}}\approx p/2$, which suggests that with the presence of many noisy features, $m_{\text{try}}$ should be larger than p/3, as in the default choice. Further investigations through data-inspired simulations and theoretical analyses are needed.

One might wonder whether the form of interaction defined by the LSS model constitutes a particularly difficult or a particularly easy form of feature interaction. In general, there appears to be no clear (mathematical) answer to this question, as one cannot define what is meant by feature interaction in a clear way for a generic (possibly discontinuous) regression function f(X)=E(Y|X). For example, it is easy to check that for any multivariate function $f:{\left[0,1\right]}^{p}->R$, one can find (possibly discontinuous) univariate functions $g,h_{1},\ldots ,h_{p}$, such that $f(x_{1},\ldots ,x_{p})=g(h_{1}(x_{1})+\ldots +h_{p}(x_{p}))$. We stress that the reason why we considered the LSS model in this work was not because it defines a particularly easy form of interaction, but rather its biological relevance, as the thresholding relationships captured in the LSS model are observed in various biological data.

Although, the LSS model is motivated from biological phenomena, some of the assumptions that we made in order to derive our theoretical results might be difficult to justify directly in real-world problems, in particular, the independence condition C1 and the nonoverlapping interaction-set condition C3. Note, however, that in many application settings, it is possible to overcome these limitations by appropriate data preprocessing—e.g., decorrelating features (recall the discussion after condition C1). Nevertheless, for future work, it will be interesting to extend our results to a general LSS model (with possibly overlapping interaction sets and correlated features) or even interaction models beyond Boolean interactions, in order to further close the gap between theory and practice.

Finally, it will also be of interest to compare LSSFind and iRF with methods that, more generally, employ an ML black-box model to extract interactions. For example, when individual features are independent, as we assume in C1, one can use Monte Carlo methods (45) to estimate higher-order Sobol indices for the fitted ML model.

## Supplementary Material

Supplementary File

## Data Availability

All source code to reproduce the simulation results and data of this paper is publicly available at GitHub, https://github.com/Yu-Group/interaction_selection. The Python iRF package which was used in the simulations is publicly available at GitHub, https://github.com/Yu-Group/iterative-Random-Forest.

## Footnotes

^*^The LSS model was first introduced by the authors of ref. 11 (including one of us) and has already been used in simulations to evaluate the performance of iRF/siRF in ref. 13.

^†^Note that ref. 15 covers the high-dimensional setting, too, but their results only depend on *s*, and not *p*.

^‡^Source code is available at GitHub (https://github.com/Yu-Group/interaction_selection).

^§^The results of iRF for the set-wise Jaccard distance in Eq. **14** are shown in *SI Appendix*, Fig. S4.

^¶^See GitHub (https://github.com/Yu-Group/iterative-Random-Forest).

^ǁ^In contrast, for the stricter performance metric Eq. **14**, which precisely captures the interaction detection property of Theorem 2, we note that LSSFind outperforms iRF; *SI Appendix*, Fig. S4.
