# Supplementary material for: Provable Boolean interaction recovery from tree ensemble obtained via random forests
Source: Proc Natl Acad Sci U S A. 2022 May 24;119(22):e2118636119. doi: 10.1073/pnas.2118636119 (PMC9295780; doi:10.1073/pnas.2118636119)
Supplement: Supplementary File [file pnas.2118636119.sapp.pdf]

Supplementary Information for

Provable Boolean Interaction Recovery from Tree Ensemble obtained via Random Forests

Merle Behr, Yu Wang, Xiao Li and Bin Yu

Bin Yu
E-mail: binyu@berkeley.edu

This PDF file includes:

Supplementary text
Figs. S1 to S5
SI References

Supporting Information Text

S1. Proof of Theorem 1 and Theorem 2

A. Proof of the population case – desirable features. Recall that there are three different sources of randomness:

1. ($\mathcal{D}$) the randomness of the data $\mathcal{D}$,
2. (T) the randomness of the tree T, given the data $\mathcal{D}$,
3. ($\mathcal{P}$) the randomness of the randomly selected path $\mathcal{P}$, given the tree T.

Note that, although the random path $\mathcal{P}$ depends on all three sources of randomness (the data randomness, the tree randomness, and the additional path randomness), when we condition on the tree T, then the random path $\mathcal{P}$ is independent of the data $\mathcal{D}$. In the first part of the proof, we will only consider the last two sources of randomness, namely, from the random tree and from the randomly selected path on the tree. Also, recall that we define $S_j^-, S_j^+ \subset [p] \times \{-1, +1\}$ as the features in $S_j \subset [p]$ with $-$ and $+$ sign, respectively, that is

$$S_j^- = \{(k, -1) \ : \ k \in S_j\} \subset [p] \times \{-1, +1\}, \quad [1]$$

$$S_j^+ = \{(k, +1) \ : \ k \in S_j\} \subset [p] \times \{-1, +1\}. \quad [2]$$

For each node t in a tree T, define $\dot{\mathrm{F}}^{\pm}(t)$ to be the set of signed features used by the parents of t in T and $\dot{\mathrm{F}}(t)$ to be the corresponding (unsigned) features. For any feature j, $(j, -)$ and $(j, +)$ can appear together in $\dot{\mathrm{F}}^{\pm}(t)$. Furthermore, let $\mathrm{F}^{\pm}(t)$ be a subset of $\dot{\mathrm{F}}^{\pm}(t)$ by only including the signed feature that corresponds to the first split of the feature if a feature appeared multiple times in the path. As a result, for any feature j, at most one of $(j, +)$ and $(j, -)$ can appear in $\mathrm{F}^{\pm}(t)$. Define $\mathrm{F}(t)$ to be the set of (unsigned) features in $\mathrm{F}^{\pm}(t)$. Because $\dot{\mathrm{F}}^{\pm}(t)$ and $\mathrm{F}^{\pm}(t)$ only differ in terms of feature signs, they correspond to the same set of features, i.e., $\dot{\mathrm{F}}(t) = \mathrm{F}(t)$. Conditioned on a tree T, at every node t of T we now define the set of *desirable* features with respect to the LSS model as follows.

Definition S1 (Desirable features)**.** *Define the desirable feature set $U(t) \subset [p]$ to be*

$$U(t) \triangleq \left\{ k \in [p] \,\middle|\, \exists\, j \in [J] \ s.t.\ k \in S_j,\ S_j^+ \cap \mathrm{F}^{\pm}(t) = \emptyset \text{ and } (k, -1) \notin \mathrm{F}^{\pm}(t) \right\}. \quad [3]$$

Note that the set of desirable features $U(t)$ at a node t is only defined w.r.t. some particular LSS model. In particular, it depends on the basic signed interactions $S_1^-, \ldots, S_J^-$. Hence, for a given tree T with node t, $U(t)$ is an oracle set, which cannot be computed from data. The way to think about $U(t)$ is that it corresponds exactly to those set of features which would yield some impurity decrease if the tree was grown by seeing the full data distribution $P(X, Y)$ and hence, making every split at the correct split point. Moreover, denote t_{leaf} to be the leaf node of $\mathcal{P}$ and we define $\mathcal{F}$ to be the desirable signed features of $F(t_{\text{leaf}})$. That is, the signed features k_t where for the node t on the path $\mathcal{P}$ we have $k_t \in U(t)$, i.e.,

$$\mathcal{F}(\mathcal{P}) \triangleq \{(k_t, b_t) \in F(t_{\text{leaf}}) \,\big|\, k_t \in U(t),\ t_{\text{leaf}} \text{ is leaf node of } \mathcal{P}\ \} \subset [p] \times \{-1, +1\}. \quad [4]$$

For notation simplicity, we use $\mathcal{F}$ as the shorthand of $\mathcal{F}(\mathcal{P})$.

Further, we define the event Ω_0 to be that the desirable features are exhausted at the leaf node:

$$\Omega_0 \triangleq \{U(t_{\text{leaf}}) = \emptyset \text{ for the leaf node } t_{\text{leaf}} \text{ of } \mathcal{P}\}. \quad [5]$$

With these definitions we get the following lemma.

Lemma S1. *For the event Ω_0 in* Eq. (5) *it holds true that*

$$\Omega_0 \subset \bigcap_{j \in [J]} \{S_j^- \subset \mathcal{F}\} \cup \{S_j^+ \cap \mathcal{F} \neq \emptyset\}, \quad [6]$$

with $\{S_j^- \subset \mathcal{F}\} \cap \{S_j^+ \cap \mathcal{F} \neq \emptyset\} = \emptyset$.

Proof. For an arbitrary interaction $j \in [J]$, it follows from the definition of $U(t)$ that Ω_0 implies either $S_j^+ \cap \mathrm{F}^{\pm}(t_{\text{leaf}}) \neq \emptyset$ or $S_j^- \subset \mathrm{F}^{\pm}(t_{\text{leaf}})$. First, consider $S_j^+ \cap \mathrm{F}^{\pm}(t_{\text{leaf}}) \neq \emptyset$. Let $(k, +1) \in S_j^+ \cap \mathrm{F}^{\pm}(t_{\text{leaf}})$ be the signed feature in $S_j^+ \cap \mathrm{F}^{\pm}(t_{\text{leaf}})$ that appears first on the path. Then, because $\mathrm{F}^{\pm}(t)$ only considers the signed features when they first appear in a path, we have that $(k, +1)$ was desirable and thus, $(k, +1) \in \mathcal{F}$, i.e., $S_j^+ \cap \mathcal{F} \neq \emptyset$. Second, consider $S_j^- \subset \mathrm{F}^{\pm}(t_{\text{leaf}})$. Then for any $(k, -1) \in S_j^-$, by definition of $F(t)$ we have that no S_j^+ feature appeared on the path before $(k, -1)$ and hence, $(k, +1) \in \mathcal{F}$, i.e., $S_j^- \subset \mathcal{F}$. Finally, recall that by definition of $\mathcal{F}$ both conditions in Eq. (6) can never happen at the same time. ☐

Moreover, define

$$C_{\text{root}}(\mathcal{D}) \triangleq \min_{k \in \cup_{j=1}^J S_j} P_T\,(\text{the root node of } T \text{ splits on feature } k \mid \mathcal{D})\,. \quad [7]$$

We state the population version of our main results below.

Theorem S1. *For all* $\tilde{S}^{\pm} \subset [p] \times \{-1, +1\}$ *with* $\tilde{s} = |\tilde{S}^{\pm}|$ *we have that almost surely*

$$P_{\mathcal{P}}\left(\tilde{S}^{\pm} \subset \mathcal{F} \;\middle|\; T, \mathcal{D}\right) \leq 0.5^{\tilde{s}} \quad [8]$$

and if $\tilde{S}^{\pm}$ *is a union signed interaction as in Definition 1 then almost surely*

$$P_{\mathcal{P}}\left(\tilde{S}^{\pm} \subset \mathcal{F} \;\middle|\; T, \mathcal{D}\right) \geq 0.5^{\tilde{s}} - P_{\mathcal{P}}\left(\Omega_0^c \mid T, \mathcal{D}\right). \quad [9]$$

Moreover, if $\tilde{S}^{\pm}$ *is not a union signed interaction then almost surely*

$$P_{(\mathcal{P},T)}\left(\tilde{S}^{\pm} \subset \mathcal{F} \;\middle|\; \mathcal{D}\right) \leq 0.5^{\tilde{s}}(1 - C_{\text{root}}(\mathcal{D})/2). \quad [10]$$

Proof of Theorem S1. Recall that the path $\mathcal{P}$ corresponding to $\mathcal{F}$ is selected in such a way: one starts at the root node t_{root} and then randomly follows the paths in the tree either to the plus $(+1)$ or to the minus (-1) direction with probability 0.5. Let $\mathcal{B}$ denote a set of i.i.d. Bernoulli coin flips taking values $+1$ and -1 with equal probability 0.5. Assume that at every node in the tree we draw one of the Bernoulli coin flips $B \in \mathcal{B}$ to decide whether we follow the path in the plus $(B = +1)$ or in the minus $(B = -1)$ direction. In particular, for any feature $k \in [p]$, let $B^k \in \mathcal{B}$ be the Bernoulli random variable we draw when k appears for the first time on $\mathcal{P}$.

Proof of Eq. (8): Note that when $(k, -1) \in \mathcal{F}$, $B^k = -1$. Similar, when $(k, +1) \in \mathcal{F}$, this implies that $B^k = +1$. Consequently, for any $\tilde{S}^{\pm} = \{(k_1, b_1), \ldots, (k_{\tilde{s}}, b_{\tilde{s}})\} \subset [p] \times \{-1, +1\}$ we have that

$$\{\tilde{S}^{\pm} \subset \mathcal{F}\} \subset \{B^{k_1} = b_1 \cap \ldots \cap B^{k_{\tilde{s}}} = b_{\tilde{s}}\} \quad [11]$$

and hence

$$P_{\mathcal{P}}\left(\tilde{S}^{\pm} \subset \mathcal{F} \middle| \, T, \mathcal{D}\right) \leq P\left(B^{k_1} = b_1 \cap \ldots \cap B^{k_{\tilde{s}}} = b_{\tilde{s}}\right) = 0.5^{\tilde{s}}. \quad [12]$$

That completes the proof.

Proof of Eq. (9): Consider any basic interaction $S_j = \{k_1, \ldots, k_{s_j}\}$, $j \in [J]$, then by Lemma S1 we have that

$$\Omega_0 \cap \{B^{k_1} = \ldots = B^{k_{s_j}} = -1\} \subset \{S_j^- \subset \mathcal{F}\}. \quad [13]$$

Moreover, when $s_j = 1$, we also have that

$$\Omega_0 \cap \{B^{k_1} = +1\} \subset \{S_j^+ \subset \mathcal{F}\}. \quad [14]$$

Consequently, when $\tilde{S}$ is a union interaction as in Definition 1 it follows that

$$\Omega_0 \cap \{B^{k_1} = b_1 \cap \ldots \cap B^{k_{\tilde{s}}} = b_{\tilde{s}}\} \subset \{\tilde{S}^{\pm} \subset \mathcal{F}\}, \quad [15]$$

which, shows Eq. (9).

Proof of Eq. (10): Assume that $\tilde{S}^{\pm}$ is not a union interaction. If any of the following is true:

- $\tilde{S}^{\pm}$ contains any noisy signed feature (k, b) that's not contained in $\cup_j S_j^+ \cup S_j^-$;
- for some signal feature $k \in \cup_j S_j$ we have that $(k, +1), (k, -1) \in \tilde{S}^{\pm}$;
- $|\tilde{S}^{\pm} \cap S_j^+| > 1$ for some $j \in [J]$;

Then by definition of $U(t)$ in Eq. (4), $P_{\mathcal{P}}\left(\tilde{S}^{\pm} \subset \mathcal{F} \middle| \, T, \mathcal{D}\right) = 0$ and thus, Eq. (10) holds.

Thus, we can assume that $\tilde{S}^{\pm}$ contains no noisy features and there exists some interaction $j \in [J]$ with $s_j > 1$ such that $(S_j^- \cup S_j^+) \cap \tilde{S}^{\pm} \neq \emptyset$ and for some $(k, -1) \in S_j^-$ we have that $(k, -1) \notin \tilde{S}^{\pm}$.

First, assume that $(k, +1) \notin \tilde{S}^{\pm}$. Then, whenever t_{root} splits on feature k, we have that $\{\tilde{S}^{\pm} \subset \mathcal{F}\}$ implies* $B^k = -1$ and thus,

$$\begin{aligned}
P_{(\mathcal{P},T)}\left(\tilde{S}^{\pm} \subset \mathcal{F} | \mathcal{D}\right) &= \sum_{\tilde{k} \in [p]} P_{(\mathcal{P},T)}\left(\tilde{S}^{\pm} \subset \mathcal{F} \cap t_{\text{root}} \text{ splits on } \tilde{k} | \mathcal{D}\right) \\
&\leq \sum_{\tilde{k} \neq k} P_{(\mathcal{P},T)}\left(B^{k_1} = b_1 \cap \ldots \cap B^{k_{\tilde{s}}} = b_{\tilde{s}} \cap t_{\text{root}} \text{ splits on } \tilde{k} | \mathcal{D}\right) \\
&+ P_{(\mathcal{P},T)}\left(B^{k_1} = b_1 \cap \ldots \cap B^{k_{\tilde{s}}} = b_{\tilde{s}} \cap B^k = -1 \cap t_{\text{root}} \text{ splits on } k | \mathcal{D}\right) \\
&= \sum_{\tilde{k} \neq k} P\left(B^{k_1} = b_1 \cap \ldots \cap B^{k_{\tilde{s}}} = b_{\tilde{s}}\right) P_T\left(t_{\text{root}} \text{ splits on } \tilde{k} | \mathcal{D}\right) \\
&+ P\left(B^{k_1} = b_1 \cap \ldots \cap B^{k_{\tilde{s}}} = b_{\tilde{s}} \cap B^k = -1\right) P_T\left(t_{\text{root}} \text{ splits on } k | \mathcal{D}\right) \\
&= 0.5^{\tilde{s}}(1 - P_T(t_{\text{root}} \text{ splits on } k | \mathcal{D})) + 0.5^{\tilde{s}+1} P_T(t_{\text{root}} \text{ splits on } k | \mathcal{D}) \\
&\leq 0.5^{\tilde{s}}(1 - C_{\text{root}}/2),
\end{aligned}$$

*Note that this requires the interactions to be disjoint, as otherwise the features in $\tilde{S}^{\pm} \cap (S_j^+ \cup S_j^-)$ may also appear in other interactions S_l with $l \neq j$ and $k \notin S_l$ and thus, even when $B^k = +1$ it is possible that $\tilde{S}^{\pm} \cap (S_j^+ \cup S_j^-) \subset \mathcal{F}$.

where we made use of the fact that the tree T is independent of the Bernoulli random variables $\mathcal{B}$.

Second, assume that $(k,+1) \in \tilde{S}^{\pm}$. If $\tilde{S}^{\pm} \cap S_j^- \neq \emptyset$, then $\{\tilde{S}^{\pm} \subset \mathcal{F}\}$ implies† that t_{root} does not split on k and thus

$$\begin{aligned} P_{(\mathcal{P},T)}\left(\tilde{S}^{\pm} \subset \mathcal{F} \middle| \mathcal{D}\right) &\leq P\left(B^{k_1} = b_1 \cap \ldots \cap B^{k_{\tilde{s}}} = b_{\tilde{s}}\right) P_T\left(t_{\text{root}} \text{ does not split on } k \middle| \mathcal{D}\right) \\ &= 0.5^{\tilde{s}} P_T\left(t_{\text{root}} \text{ does not split on } k \middle| \mathcal{D}\right) \leq 0.5^{\tilde{s}}(1 - C_{\text{root}}). \end{aligned}$$

If $\tilde{S}^{\pm} \cap S_j^- = \emptyset$, let $k^\star \in S_j$ and $k^\star \neq k$. Because $(k,+1) \in \tilde{S}^{\pm}$, we can assume that $(k^\star,+1) \notin \tilde{S}^{\pm}$; otherwise $|\tilde{S}^{\pm} \cap S_j^+| > 1$, which implies $P\left(\tilde{S}^{\pm} \subset \mathcal{F}\right) = 0$. When t_{root} splits on $k^\star$, $\{\tilde{S}^{\pm} \subset \mathcal{F}\}$ implies‡ $B^{k^\star} = -1$ and thus,

$$\begin{aligned} P_{(\mathcal{P},T)}\left(\tilde{S}^{\pm} \subset \mathcal{F} \middle| \mathcal{D}\right) &= \sum_{\tilde{k} \in [p]} P_{(\mathcal{P},T)}\left(\tilde{S}^{\pm} \subset \mathcal{F} \cap t_{\text{root}} \text{ splits on } \tilde{k} \middle| \mathcal{D}\right) \\ &\leq \sum_{\tilde{k} \neq k^\star} P_{(\mathcal{P},T)}\left(B^{k_1} = b_1 \cap \ldots \cap B^{k_{\tilde{s}}} = b_{\tilde{s}} \cap t_{\text{root}} \text{ splits on } \tilde{k} \middle| \mathcal{D}\right) \\ &+ P_{(\mathcal{P},T)}\left(B^{k_1} = b_1 \cap \ldots \cap B^{k_{\tilde{s}}} = b_{\tilde{s}} \cap B^{k^\star} = -1 \cap t_{\text{root}} \text{ splits on } k^\star \middle| \mathcal{D}\right) \\ &\leq 0.5^{\tilde{s}}(1 - P_T(t_{\text{root}} \text{ splits on } k^\star | \mathcal{D})) + 0.5^{\tilde{s}+1} P_{(\mathcal{P},T)}(t_{\text{root}} \text{ splits on } k^\star | \mathcal{D}) \\ &\leq 0.5^{\tilde{s}}(1 - C_{\text{root}}/2). \end{aligned}$$

Thus, we have shown Eq. (10). □

B. Proof of the finite sample case.

B.1. Filtering of desirable features and impurity. Recall that $R_{t,l} = R_t \cap \{X | X_{k_t} \leq \gamma_t\}$ and $R_{t,r} = R_t \cap \{X | X_{k_t} > \gamma_t\}$ denote the region corresponding to the left and right children for node t. In other words, node t divides the region R_t into $R_{t,l}$ and $R_{t,r}$. Recall that $N_n(t)$ is the number of samples in the region R_t, i.e., $N_n(t) = \sum_{i=1}^n \mathbf{1}(x_i \in R_t)$. We will use an equivalent formula for the impurity as in Lemma S2.

Lemma S2. $\Delta_I^n(R_{t,l}, R_{t,r})$ *defined in* Eq. (6) *in the main paper is equivalent to* Eq. (16)*:*

$$\Delta_I^n(R_{t,l}, R_{t,r}) = \frac{N_n(t_l) N_n(t_r)}{n(N_n(t_l) + N_n(t_r))} \left(\frac{1}{N_n(t_l)} \sum_{\mathbf{x}_i \in R_{t,l}} y_i - \frac{1}{N_n(t_r)} \sum_{\mathbf{x}_i \in R_{t,r}} y_i \right)^2. \qquad [16]$$

Proof. We have that

$$\begin{aligned} &\Delta_I^n(R_{t,l}, R_{t,r}) \\ =&\frac{1}{n}\left(\sum_{\mathbf{x}_i \in R_t} (y_i - \frac{1}{N_n(t)} \sum_{\mathbf{x}_i \in R_t} y_i)^2 - \sum_{\mathbf{x}_i \in R_{t,l}} (y_i - \frac{1}{N_n(t_l)} \sum_{\mathbf{x}_i \in R_{t,l}} y_i)^2 - \sum_{\mathbf{x}_i \in R_{t,r}} (y_i - \frac{1}{N_n(t_r)} \sum_{\mathbf{x}_i \in R_{t,r}} y_i)^2 \right) \\ =&\frac{1}{n}\left(\sum_{\mathbf{x}_i \in R_t} y_i^2 - \frac{1}{N_n(t)} (\sum_{\mathbf{x}_i \in R_t} y_i)^2 - \sum_{\mathbf{x}_i \in R_{t,l}} y_i^2 + \frac{1}{N_n(t_l)} (\sum_{\mathbf{x}_i \in R_{t,l}} y_i)^2 - \sum_{\mathbf{x}_i \in R_{t,r}} y_i^2 + \frac{1}{N_n(t_r)} (\sum_{\mathbf{x}_i \in R_{t,r}} y_i)^2 \right) \\ =&\frac{1}{n}\left(-\frac{1}{N_n(t)} (\sum_{\mathbf{x}_i \in R_t} y_i)^2 + \frac{1}{N_n(t_l)} (\sum_{\mathbf{x}_i \in R_{t,l}} y_i)^2 + \frac{1}{N_n(t_r)} (\sum_{\mathbf{x}_i \in R_{t,r}} y_i)^2 \right). \end{aligned}$$

If we denote $A = \sum_{\mathbf{x}_i \in R_{t,l}} y_i$ and $B = \sum_{\mathbf{x}_i \in R_{t,r}} y_i$, the above formula is the same as :

$$\begin{aligned} &\frac{1}{n}\left(-\frac{1}{N_n(t)}(A+B)^2 + \frac{1}{N_n(t_l)} A^2 + \frac{1}{N_n(t_r)} B^2 \right) \\ =&\frac{1}{n}\left(\frac{N_n(t_r)}{N_n(t_l) N_n(t)} A^2 + \frac{N_n(t_l)}{N_n(t_r) N_n(t)} B^2 - \frac{2}{N_n(t)} AB \right) \\ =&\frac{N_n(t_l) N_n(t_r)}{n N_n(t)} \left(\frac{1}{N_n(t_l)^2} A^2 + \frac{1}{N_n(t_r)^2} B^2 - \frac{2}{N_n(t_l) N_n(t_r)} AB \right) = \frac{N_n(t_l) N_n(t_r)}{n N_n(t)} \left(\frac{1}{N_n(t_l)} A - \frac{1}{N_n(t_r)} B \right)^2. \end{aligned}$$

□

† Again, this requires the interactions to be disjoint, as otherwise the features in $\tilde{S}^{\pm} \cap (S_j^- \cup S_j^+) \setminus (k,+1)$ may also appear in other interactions S_l with $l \neq j$ and thus, even when t_{root} splits on k with $B^k = +1$, it is possible that $\tilde{S}^{\pm} \cap (S_j^- \cup S_j^+) \setminus (k,+1) \subset \mathcal{F}$.

‡ Again, this requires the interactions to be disjoint.

Let $\mathscr{R}$ denote the set of axis-aligned hyper-rectangles obtained by splitting the unit hyper-rectangle consecutively, where each split satisfies assumption A2 in the main text. We study $\mathscr{R}$ because it contains all the possible rectangles that can represent region of a node in a tree. Let $\mathscr{R}_d$ be the set of rectangles obtained by splitting the unit hyper-rectangle d times, where each split satisfies assumption A2 from the main text. Then $\mathscr{R} = \cup_{d\geq 1}\mathscr{R}_d$, and for any $R \in \mathscr{R}_d$, we have $\mu(R) \leq (1 - C_\gamma)^d$ (recall that $\mu(R)$ denotes the volume of R). For any region R, we denote N_R to be the number of points in R.

Lemma S3. *Suppose that assumption A2 from the main text is satisfied. Then for any $d \geq 1$ it holds true that*

$$\max_{R\in\cup_{d_1>d}\mathscr{R}_{d_1}} \left|\frac{1}{n}\sum_{i=1}^n y_i \mathbf{1}(\mathbf{x}_i \in R_1) - \mathbb{E}(Y\cdot \mathbf{1}(X\in R))\right| \leq C_Y \left(\max_{R\in\mathscr{R}_d}\left|\frac{N_R}{n} - \mu(R)\right|\right) + 2C_Y(1-C_\gamma)^d.$$

Proof of Lemma. For any $R_1 \in \mathscr{R}_{d_1}$, $d_1 > d$, there exists $R_0 \in \mathscr{R}_d$ such that $R_1 \subset R_0$. Therefore, $N_{R_1} < N_{R_0}$ and

$$\left|\frac{1}{n}\sum_{i=1}^n y_i \mathbf{1}(\mathbf{x}_i \in R_1)\right| \leq \frac{N_{R_1}}{n}C_Y < \frac{N_{R_0}}{n}C_Y.$$

Since $R_0 \in \mathscr{R}_d$, we have

$$\frac{N_{R_0}}{n} \leq \max_{R\in\mathscr{R}_d}\left|\frac{N_R}{n} - \mu(R)\right| + \max_{R\in\mathscr{R}_d}\mu(R) \leq \max_{R\in\mathscr{R}_d}\left|\frac{N_R}{n} - \mu(R)\right| + (1-C_\gamma)^d. \quad [17]$$

Therefore,

$$\begin{aligned}
&\left|\frac{1}{n}\sum_{i=1}^n y_i \mathbf{1}(\mathbf{x}_i \in R_1) - \mathbb{E}(Y\cdot \mathbf{1}(X\in R))\right| \\
\leq\ & \left|\frac{1}{n}\sum_{i=1}^n y_i \mathbf{1}(\mathbf{x}_i \in R_1)\right| + \left|\mathbb{E}(Y\cdot \mathbf{1}(X\in R))\right| \\
\leq\ & \frac{N_{R_0}}{n}C_Y + C_Y(1-C_\gamma)^{d+1} \\
\leq\ & C_Y\left(\max_{R\in\mathscr{R}_d}\left|\frac{N_R}{n} - \mu(R)\right| + (1-C_\gamma)^d\right) + C_Y(1-C_\gamma)^{d+1} \\
\leq\ & C_Y\left(\max_{R\in\mathscr{R}_d}\left|\frac{N_R}{n} - \mu(R)\right|\right) + 2C_Y(1-C_\gamma)^d.
\end{aligned}$$

Since R_1 is arbitrary, we have

$$\max_{R\in\cup_{d_1>d}\mathscr{R}_{d_1}} \left|\frac{1}{n}\sum_{i=1}^n y_i \mathbf{1}(\mathbf{x}_i \in R_1) - \mathbb{E}(Y\cdot \mathbf{1}(X\in R))\right| \leq C_Y \left(\max_{R\in\mathscr{R}_d}\left|\frac{N_R}{n} - \mu(R)\right|\right) + 2C_Y(1-C_\gamma)^d. \quad [18]$$

□

Proposition S4. *Suppose that constraint C4 and assumption A2 from the main text hold true. Then*

$$\max_{R\in\mathscr{R}}\left|\frac{N_R}{n} - \mu(R)\right| \xrightarrow{p} 0,$$

and

$$\max_{R\in\mathscr{R}}\left|\frac{1}{n}\sum_{i=1}^n y_i \mathbf{1}(\mathbf{x}_i \in R) - \mathbb{E}(Y\cdot \mathbf{1}(X\in R))\right| \xrightarrow{p} 0.$$

Proof. For any fixed d, let $G_n(\mathscr{R}_d)$ be the growth function for the set of rectangles $\mathscr{R}_d$ defined in Chapter 5.2 of Vapnik (1), i.e.,

$$G_n(\mathscr{R}_d) \triangleq \max_{\mathbf{x}_i\in\mathbb{R}^p, y_i\in\mathbb{R}} \log\left|\left\{(\mathbf{1}(y_1\geq\theta, \mathbf{x}_1\in R), \ldots, \mathbf{1}(y_n\geq\theta, \mathbf{x}_n\in R))\middle| R\in\mathscr{R}_d, \theta\in\mathbb{R}\right\}\right|.$$

Here for any set A, $|A|$ denotes the number of elements in A.

We claim that $G_n(\mathscr{R}_d) \leq \log(n(2np)^d)$. This is because at each of d splits, we have at most p directions and at most n split points to choose from. Therefore, splitting d times can create no more than $(2np)^d$ different separations of the n data points. Furthermore, within each rectangle, the indicator functions $\mathbf{1}(y_i \geq \theta), \theta \in \mathbb{R}$ can at most create n separations.

Thus,

$$G_n(\cup_{d_0\leq d}\mathscr{R}_{d_0}) \leq \log(d\exp(G_n(\mathscr{R}_d))) \leq \log(nd(2np)^d), \quad [19]$$

and

$$\frac{G_n(\cup_{d_0\leq d}\mathscr{R}_{d_0})}{n} \leq \frac{\log(nd) + d\log(2n)}{n} + \frac{d\log p}{n} \to 0.$$

Therefore, by Theorem 5.1 of (1):

Theorem 5.1 in (1): Let $A \leq Q(z,\alpha) \leq B$, $\alpha \in \Lambda$ be a measurable set of bounded real-valued functions. Let G_n be the growth function of the indicator functions induced by Q, then we have the following inequality:

$$P\left\{\sup_{\alpha\in\Lambda}\left(\int Q(z,\alpha)dF(z) - \frac{1}{n}\sum_{i=1}^{n} Q(z_i,\alpha)\right) > \epsilon\right\} \leq 4\exp\left\{\left(\frac{G_{2n}}{n} - \frac{(\epsilon - n^{-1})^2}{(B-A)^2}\right)n\right\}.$$

we have

$$\max_{R\in\cup_{d_0\leq d}\mathscr{R}_{d_0}}\left|\frac{1}{n}\sum_{i=1}^{n} y_i \mathbf{1}(\mathbf{x}_i \in R) - \mathbb{E}(Y\cdot\mathbf{1}(X\in R))\right| \xrightarrow{p} 0. \quad [20]$$

Taking $Y = 1$ and we have

$$\max_{R\in\cup_{d_0\leq d}\mathscr{R}_{d_0}}\left|\frac{N_R}{n} - \mu(R)\right| \xrightarrow{p} 0. \quad [21]$$

By Lemma S3 and the above equation, we have

$$\begin{aligned}&\max_{R\in\cup_{d_1>d}\mathscr{R}_{d_1}}\left|\frac{1}{n}\sum_{i=1}^{n} y_i\mathbf{1}(\mathbf{x}_i\in R_1) - \mathbb{E}(f(X)\cdot\mathbf{1}(X\in R_1))\right| \\ &\leq C_Y\left(\max_{R\in\mathscr{R}_d}\left|\frac{N_R}{n} - \mu(R)\right|\right) + 2C_Y(1-C_\gamma)^d \overset{p}{\lesssim} 3C_Y(1-C_\gamma)^d.\end{aligned} \quad [22]$$

Since that holds for any fixed $d > 0$, we know the left hand side of Eq. (22) converges to zero in probability. Combining Eq. (20) and Eq. (22), we have shown that:

$$\max_{R\in\mathscr{R}}\left|\frac{1}{n}\sum_{i=1}^{n} y_i\mathbf{1}(\mathbf{x}_i\in R) - \mathbb{E}(Y\cdot\mathbf{1}(X\in R))\right| \xrightarrow{p} 0.$$

Since this holds for any bounded random variable Y, we can take $Y = 1$ and we have shown

$$\max_{R\in\mathscr{R}}\left|\frac{N_R}{n} - \mu(R)\right| \xrightarrow{p} 0. \quad [23]$$

That completes the proof. □

Proposition S5 (Subgaussian case)**.** *Suppose that assumption A2 from the main text holds true and* $(\log n)^{1+\delta}\log p/n \to 0$ *for some* $\delta > 0$*. Suppose* $Y = E(Y|X) + Z$ *where* Z *is independent of* X *and* 1*-subgaussian. Then*

$$\max_{R\in\mathscr{R}}\left|\frac{1}{n}\sum_{i=1}^{n} y_i\mathbf{1}(\mathbf{x}_i\in R) - \mathbb{E}(Y\cdot\mathbf{1}(X\in R))\right| \xrightarrow{p} 0.$$

Proof. Denote $f(X) = E(Y|X)$ and $C_Y = \sum_{j=0}^{J}|\beta_j|$. Then $|f(X)| \leq C_Y$. Note that

$$\begin{aligned}&\max_{R\in\mathscr{R}}\left|\frac{1}{n}\sum_{i=1}^{n} y_i\mathbf{1}(\mathbf{x}_i\in R) - \mathbb{E}(Y\cdot\mathbf{1}(X\in R))\right| \\ \leq\ &\max_{R\in\mathscr{R}}\left|\frac{1}{n}\sum_{i=1}^{n} f(\mathbf{x}_i)\mathbf{1}(\mathbf{x}_i\in R) - \mathbb{E}(f(X)\cdot\mathbf{1}(X\in R))\right| + \max_{R\in\mathscr{R}}\left|\frac{1}{n}\sum_{i=1}^{n} z_i\mathbf{1}(\mathbf{x}_i\in R)\right|.\end{aligned}$$

Here $z_i = y_i - f(\mathbf{x}_i)$ represents the noise terms. Our proof proceeds in the following two steps.

Step 1. Show that

$$\max_{R\in\mathscr{R}}\left|\frac{1}{n}\sum_{i=1}^{n} f(\mathbf{x}_i)\mathbf{1}(\mathbf{x}_i\in R) - \mathbb{E}(f(X)\cdot\mathbf{1}(X\in R))\right| \xrightarrow{p} 0. \quad [24]$$

Step 1 is similar to the proof of Proposition S4 but the difference is that we need the convergence rate. Let

$$\delta_0 = \frac{\delta}{2\delta+4},$$

and take

$$d = \left(\frac{n}{\log(np)}\right)^{\delta_0} \to \infty.$$

Let $G_n(\mathscr{R}_d)$ be the growth function for the set of rectangles $\mathscr{R}_d$. By Eq. (19), we have

$$\frac{G_n(\cup_{d_0\le d}\mathscr{R}_{d_0})}{n} \le \frac{\log(nd)+d\log(2n)}{n} + \frac{d\log p}{n} = O\left(\frac{d\log(np)}{n}\right) = O\left(\left(\frac{\log(np)}{n}\right)^{1-\delta_0}\right) \to 0.$$

Therefore, by Theorem 5.1 of (1), we have

$$\max_{R\in\cup_{d_0\le d}\mathscr{R}_{d_0}} \left|\frac{1}{n}\sum_{i=1}^n f(\mathbf{x}_i)\mathbf{1}(\mathbf{x}_i \in R_1) - \mathbb{E}(f(X)\cdot \mathbf{1}(X\in R))\right| = o\left(\left(\frac{\log(np)}{n}\right)^{1/2-\delta_0}\right) \xrightarrow{p} 0. \quad [25]$$

Since this holds for any bounded random variable Y, we can take $Y=1$ and it follows that

$$\max_{R\in\mathscr{R}_d}\left|\frac{N_R}{n} - \mu(R)\right| \le \max_{R\in\cup_{d_0\le d}\mathscr{R}_{d_0}}\left|\frac{N_R}{n} - \mu(R)\right| = o\left(\left(\frac{\log(np)}{n}\right)^{1/2-\delta_0}\right) \xrightarrow{p} 0. \quad [26]$$

Since $d\to\infty$, $(1-C_\gamma)^d \to 0$. Therefore, by Lemma S3, we have

$$\max_{R\in\cup_{d_1>d}\mathscr{R}_{d_1}} \left|\frac{1}{n}\sum_{i=1}^n f(\mathbf{x}_i)\mathbf{1}(\mathbf{x}_i \in R_1) - \mathbb{E}(f(X)\cdot \mathbf{1}(X\in R))\right| \xrightarrow{p} 0. \quad [27]$$

Combining Eq. (25) and Eq. (27), Eq. (24) is proved.

Step 2. Show that

$$\max_{R\in\mathscr{R}}\left|\frac{1}{n}\sum_{i=1}^n z_i\mathbf{1}(\mathbf{x}_i\in R)\right| \xrightarrow{p} 0. \quad [28]$$

Note that

$$\max_{R\in\mathscr{R}}\left|\frac{1}{n}\sum_{i=1}^n z_i\mathbf{1}(\mathbf{x}_i\in R)\right| = \max\left\{\max_{R\in\cup_{d_0\le d}\mathscr{R}_{d_0}}\left|\frac{1}{n}\sum_{i=1}^n z_i\mathbf{1}(\mathbf{x}_i\in R)\right|, \max_{R\in R\in\cup_{d_1>d}\mathscr{R}_{d_1}}\left|\frac{1}{n}\sum_{i=1}^n z_i\mathbf{1}(\mathbf{x}_i\in R)\right|\right\}.$$

Therefore, it suffices to prove that both of the two terms on the right hand size converges to 0 in probability. We begin with the first term: $\max_{R\in\cup_{d_0\le d}\mathscr{R}_{d_0}}\left|\frac{1}{n}\sum_{i=1}^n z_i\mathbf{1}(\mathbf{x}_i\in R)\right|$. Since X and Z are independent and Z is 1-subgaussian, by Hoeffding inequality,

$$P\left(\left|\frac{1}{n}\sum_{i=1}^n z_i\mathbf{1}(\mathbf{x}_i\in R)\right| \ge \epsilon/2 \;\middle|\; X\right) = P\left(\left|\frac{1}{n}\sum_{i=1}^{N_R} z_i\right| \ge \epsilon/2\right) \le 2\exp\left(-\frac{n^2\epsilon^2}{8N_R}\right)$$

for any rectangle R. Therefore by union bound,

$$\begin{aligned}
&P\left(\max_{R\in\cup_{d_0\le d}\mathscr{R}_{d_0}}\left|\frac{1}{n}\sum_{i=1}^n z_i\mathbf{1}(\mathbf{x}_i\in R)\right| \ge \epsilon/2 \;\middle|\; X\right)\\
&\le 2\exp(G_n(\cup_{d_0\le d}\mathscr{R}_{d_0}))\exp\left(-\frac{n\epsilon^2}{8}\right)\\
&\le 2\exp\left(\log(nd(2np)^d) - \frac{n\epsilon^2}{8}\right) \to 0
\end{aligned}$$

for any $\epsilon>0$. Since the above upper bound on the probability is independent of X, we conclude that

$$\max_{R\in\cup_{d_0\le d}\mathscr{R}_{d_0}}\left|\frac{1}{n}\sum_{i=1}^n z_i\mathbf{1}(\mathbf{x}_i\in R)\right| \xrightarrow{p} 0.$$

We now turn to the second term $\max_{R\in R\in\cup_{d_1>d}\mathscr{R}_{d_1}}\left|\frac{1}{n}\sum_{i=1}^n z_i\mathbf{1}(\mathbf{x}_i\in R)\right|$. Let $\mathscr{R}^{s_0}$ be the set of rectangles with at most $s_0 = n/(\log n)^{1/2+\delta_0}$ samples, then $\log|\mathscr{R}^{s_0}| \le (s_0+1)\log n$. By union bound,

$$\begin{aligned}
&P\left(\max_{R\in\mathscr{R}^{s_0}}\left|\frac{1}{n}\sum_{i=1}^n z_i\mathbf{1}(\mathbf{x}_i\in R)\right| \ge \epsilon/2 \;\middle|\; X\right)\\
&\le 2\exp(\log|\mathscr{R}^{s_0}|)\exp\left(-\frac{n\epsilon^2}{8}\right)\\
&\le 2\exp\left((s_0+1)\log n - \frac{n^2\epsilon^2}{8s_0}\right) \to 0.
\end{aligned}$$

Therefore,

$$\max_{R\in\mathscr{R}^{s_0}}\left|\frac{1}{n}\sum_{i=1}^{n} z_i\mathbf{1}(\mathbf{x}_i\in R)\right|\to 0.$$

Hence, to prove Eq. (28), it suffices to show that $\cup_{d_1>d}\mathscr{R}_{d_1}\subset\mathscr{R}^{s_0}$ with probability tending to 1. Note that by definition of δ_0, $\frac{1/2+\delta_0}{1/2-\delta_0}=1+\delta$. Therefore

$$\left(\frac{\log(np)}{n}\right)^{\frac{1}{2}-\delta_0}(\log n)^{\frac{1}{2}+\delta_0}=\left(\frac{\log(np)(\log n)^{1+\delta}}{n}\right)^{\frac{1}{2}-\delta_0}=\left(\frac{(\log n)^{2+\delta}+\log p(\log n)^{1+\delta}}{n}\right)^{\frac{1}{2}-\delta_0}\to 0.$$

By Eq. (17) and Eq. (26) we have

$$\max_{R\in\cup_{d_1>d}\mathscr{R}_{d_1}} N_R\le\max_{R\in\mathscr{R}_d} N_R=o\left(n\left(\frac{\log(np)}{n}\right)^{1/2-\delta_0}\right)=o(s_0).$$

Therefore, $\max_{R\in\cup_{d_1>d}\mathscr{R}_{d_1}} N_R\le s_0$ with probability tending to 1. The proof is now complete. □

Define population impurity decrease $\Delta_I(t)$ at a node t to be

$$\Delta_I(t)=\mathrm{Var}(Y|R_t)-\frac{\mu(R_{t_l})}{\mu(R_t)}\mathrm{Var}(Y|R_{t_l})-\frac{\mu(R_{t_r})}{\mu(R_t)}\mathrm{Var}(Y|R_{t_r}).\qquad [29]$$

Similar to Lemma S2, we know it is equivalent to:

$$\Delta_I(R_{t,l}(\gamma;k),R_{t,r}(\gamma;k))=\frac{\mu(R_{t,l}(\gamma;k))\mu(R_{t,r}(\gamma;k))}{\mu(R_t(\gamma;k))}\Big[\mathbb{E}(Y|X\in R_{t,l}(\gamma;k))-\mathbb{E}(Y|X\in R_{t,r}(\gamma;k))\Big]^2.\qquad [30]$$

The following proposition shows that the finite-sample impurity decrease converges to the population impurity decrease uniformly.

Proposition S6. *Suppose that constraint C4 and assumption A2 from the main text are satisfied. Then, we have the following two uniform convergence results:*

a. $\max_{R\in\mathscr{R}}\left|\frac{N_R}{n}-\mu(R)\right|\xrightarrow{p}0,$

b. $\sup_{R_{t,l},R_{t,r}\in\mathscr{R}}\left|\Delta_I^n(R_{t,l},R_{t,r})-\Delta_I(R_{t,l},R_{t,r})\right|\xrightarrow{p}0.$

Proof. **a.** This follow directly from Proposition S4.

b. Let $f(x_1,x_2,y_1,y_2)=\frac{x_1x_2}{x_1+x_2}(y_1-y_2)^2$. Then f is a Lipschitz function on $[0,1]\times[0,1]\times[-C_Y-1,C_Y+1]\times[-C_Y-1,C_Y+1]$. Use the fact that $\max_{R\in\mathscr{R}}\left|\frac{1}{n}\sum_{i=1}^n y_i\mathbf{1}(\mathbf{x}_i\in R)-\mathbb{E}(Y\cdot\mathbf{1}(X\in R))\right|\xrightarrow{p}0$ in Proposition S4 and the fact $\max_{R\in\mathscr{R}}\left|\frac{N_R}{n}-\mu(R)\right|\xrightarrow{p}0$ in a., by the continuous mapping theorem, we have

$$\sup_{R_{t,l},R_{t,r}\in\mathscr{R}}\left|\Delta_I^n(R_{t,l},R_{t,r})-\Delta_I(R_{t,l},R_{t,r})\right|\xrightarrow{p}0.$$

□

Now we analyze the impurity decrease at each node of a tree. We consider three families of trees: $\mathcal{T}_0$, $\mathcal{T}_1$ and $\mathcal{T}_2$:

$$\mathcal{T}_0\triangleq\{\text{Any tree that satisfies A2}\}.$$

$$\mathcal{T}_1\triangleq\{\text{Any CART tree that satisfies A2 and A4}\}.$$

$$\mathcal{T}_2\triangleq\{\text{Any CART tree that satisfies A2, A4, and A3}\}.$$

$\mathcal{T}_1$ is the family of CART trees that satisfy our assumptions but m_{try} can be arbitrary. $\mathcal{T}_1$ is more restricted than $\mathcal{T}_0$ in the sense that the threshold γ_t of any node t of any tree in $\mathcal{T}_1$ must maximize the finite sample impurity decrease in Eq. (6). Thus, $\mathcal{T}_1$ depends on the data. For any $T\in\mathcal{T}_0$ and any $t\in T$ such that $\mathring{U}(t)\neq\emptyset$, its region R_t is a rectangle:

$$R_t=\{x\in\mathbb{R}^p|\forall\ell\in[p],c_{low,\ell}<x_\ell\le c_{high,\ell}\}.\qquad [31]$$

where $c_{low,\ell},c_{high,\ell}\in[0,1]$.

By the definition of desirable feature set $U(t)$ in Eq. (3), we have its equivalent formula:

$$U(t)\triangleq\cup_{j\in[J]:S_j^{+}\cap\mathrm{F}^{\pm}(t)=\emptyset}S_j/\mathrm{F}(t).$$

Define the set of noisy features to be its complement: $[p]/U(t)$. We also define

$$\mathring{U}(t) \triangleq \cup_{j\in[J]:S_j^+\cap\dot{\mathrm{F}}^\pm(t)=\emptyset} S_j/\mathrm{F}(t).$$

Since $\mathrm{F}^\pm(t) \subset \dot{\mathrm{F}}^\pm(t)$, $\mathring{U}(t) \subset U(t)$. For any γ, denote $R_{t,l}(\gamma;k) = R_t \cap \{X|X_k \le \gamma\}$ and $R_{t,r}(\gamma;k) = R_t \cap \{X|X_k > \gamma\}$. First, for any node $t \in T$ and any $k \in \mathring{U}(t)$, we have a characterization for the impurity decrease:

Lemma S7. *For any $T \in \mathcal{T}_0$, $t \in T$, $j \in [J]$, $k \in S_j \cap U(t)$, and $\gamma \in (0,1)$,*

$$\begin{aligned}
&\Delta_I(R_{t,l}(\gamma;k), R_{t,r}(\gamma;k)) \\
=&\mu(R_t)\cdot\beta_j^2 P(\forall\, \ell \in S_j/\{k\},\ X_\ell \le \gamma_\ell | X \in R_t)^2 \cdot \left(\mathbf{1}(\gamma \le \gamma_k)\cdot\frac{(1-\gamma_k)^2\gamma}{(1-\gamma)} + \mathbf{1}(\gamma > \gamma_k)\cdot\frac{\gamma_k^2(1-\gamma)}{\gamma}\right).
\end{aligned}$$

Proof of Lemma S7. Since $k \in U(t)$, we know that k is not in $\mathrm{F}(t)$. That means any of t's parents do not split on k. In other words, R_t does not have any constraints for feature k, i.e., $c_{low,k} = 0$ and $c_{high,k} = 1$. Thus, we know that

$$\mu(R_{t,l}(\gamma;k)) = \mu(R_t)\cdot\gamma \qquad [32]$$

and

$$\mu(R_{t,r}(\gamma;k)) = \mu(R_t)\cdot(1-\gamma). \qquad [33]$$

Recall that Δ_I in Eq. (29) has its equivalent formula Eq. (30):

$$\Delta_I(R_{t,l}(\gamma;k), R_{t,r}(\gamma;k)) = \frac{\mu(R_{t,l}(\gamma;k))\mu(R_{t,r}(\gamma;k))}{\mu(R_t(\gamma;k))}\Big[\mathbb{E}(Y|X\in R_{t,l}(\gamma;k)) - \mathbb{E}(Y|X\in R_{t,r}(\gamma;k))\Big]^2$$

where the conditional expectations are

$$\mathrm{E}(Y|X\in R_{t,l}(\gamma;k)) = \sum_{j'=1}^{J}\beta_{j'}P\left(\forall \ell\in S_{j'},\ X_\ell\le\gamma_\ell \middle| X\in R_{t,l}(\gamma;k)\right), \qquad [34]$$

and

$$\mathrm{E}(Y|X\in R_{t,r}(\gamma;k)) = \sum_{j'=1}^{J}\beta_{j'}P\left(\forall \ell\in S_{j'},\ X_\ell\le\gamma_\ell \middle| X\in R_{t,r}(\gamma;k)\right). \qquad [35]$$

Now we will analyze Eq. (34) and Eq. (35). To ease the notations, we define the following three events:

$$\begin{aligned}
A_{j'} =&\{X_\ell \le \gamma_\ell,\ \forall \ell \in S_{j'}\}, && [36]\\
B =&\{X \in R_t\}, && [37]\\
C_k =&\{X_k \le \gamma\}. && [38]
\end{aligned}$$

Then Eq. (34) becomes $\sum_{j'=1}^{J}\beta_{j'}P(A_{j'}|BC_k)$. Because R_t has no constraints on k, B does not involve feature k. When $j' \ne j$ (namely, $k \notin S_{j'}$), $A_{j'}$ also does not involve feature k. Thus, C_k is independent of $(A_{j'}, B)$, which implies $P(A_{j'}|BC_k) = \frac{P(A_{j'}BC_k)}{P(BC_k)} = \frac{P(A_{j'}B)P(C_k)}{P(B)P(C_k)} = P(A_{j'}|B)$. Similarly this holds for Eq. (35). Therefore, when $j' \ne j$:

$$P\left(\forall \ell\in S_{j'},\ X_\ell\le\gamma_\ell \middle| X\in R_{t,l}(\gamma;k)\right) = P\left(\forall \ell\in S_{j'},\ X_\ell\le\gamma_\ell \middle| X\in R_{t,r}(\gamma;k)\right).$$

When $j' = j$,

$$\begin{aligned}
&P\left(\forall \ell\in S_{j},\ X_\ell\le\gamma_\ell \middle| X\in R_{t,l}(\gamma;k)\right) - P\left(\forall \ell\in S_{j},\ X_\ell\le\gamma_\ell \middle| X\in R_{t,r}(\gamma;k)\right)\\
(X_k \text{ is ind. of } X_\ell \text{ for } \ell\ne k) =&P(\forall\, \ell\in S_j/\{k\},\ X_\ell\le\gamma_\ell|X\in R_t)\cdot\\
&\Big(P(X_k\le\gamma_k|X_k\le\gamma) - P(X_k\le\gamma_k|X_k>\gamma)\Big)\\
=&P(\forall\, \ell\in S_j/\{k\},\ X_\ell\le\gamma_\ell|X\in R_t)\cdot\\
&\left(\mathbf{1}(\gamma\le\gamma_k)\cdot\frac{1-\gamma_k}{1-\gamma} + \mathbf{1}(\gamma>\gamma_k)\cdot\frac{\gamma_k}{\gamma}\right).
\end{aligned}$$

Therefore, Eq. (30) becomes:

$$\begin{aligned}
&\frac{\mu(R_{t,l}(\gamma;k))\mu(R_{t,r}(\gamma;k))}{\mu(R_t)}\Big(\mathbb{E}(Y|X\in R_{t,l}(\gamma;k))-\mathbb{E}(Y|X\in R_{t,r}(\gamma;k))\Big)^2\\
&=\mu(R_t)\gamma(1-\gamma)\cdot\beta_j^2P(\forall\ \ell\in S_j/\{k\},\ X_\ell\le\gamma_\ell|X\in R_t)^2\cdot\\
&\qquad\Big(\mathbf{1}(\gamma\le\gamma_k)\cdot\frac{(1-\gamma_k)^2}{(1-\gamma)^2}+\mathbf{1}(\gamma>\gamma_k)\cdot\frac{\gamma_k^2}{\gamma^2}\Big)\\
&=\mu(R_t)\cdot\beta_j^2P(\forall\ \ell\in S_j/\{k\},\ X_\ell\le\gamma_\ell|X\in R_t)^2\cdot\\
&\qquad\Big(\mathbf{1}(\gamma\le\gamma_k)\cdot\frac{(1-\gamma_k)^2\gamma}{(1-\gamma)}+\mathbf{1}(\gamma>\gamma_k)\cdot\frac{\gamma_k^2(1-\gamma)}{\gamma}\Big).
\end{aligned}$$

160 That completes the proof. □

161 **Lemma S8.** *For $T\in\mathcal{T}_0$, $t\in T$, if there exists $j\in[J]$ and $k\in S_j$ such that $k\in\mathring{U}(t)$, then*

162 $$P(\forall\ \ell\in S_j/\{k\},\ X_\ell\le\gamma_\ell|X\in R_t)\ge C_\gamma^{s_j-1}.$$

Proof of Lemma S8. Because $k\in S_j$ and $k\in\mathring{U}(t)$, we know that $S_j^+\cap\dot{\mathrm{F}}^\pm(t)=\emptyset$. That means node t is not at the right branch of any node that splits on features in S_j. Thus,

$$c_{low,\ell}=0 \text{ when } \ell\in S_j. \qquad [39]$$

Also, $c_{high,k}=1$ and $c_{low,k}=0$ because $k\in\mathring{U}(t)$. Then, $P(\forall\ \ell\in S_j/\{k\},\ X_\ell\le\gamma_\ell|X\in R_t)$ is

$$\begin{aligned}
&\frac{P(\forall\ell\in S_j/\{k\}\ X_\ell\le\gamma_\ell,X\in R_t)}{\mu(R_t)}\\
\text{(Due to Eq. (39))}=&\frac{\prod_{\ell\in[p]/S_j}(c_{high,\ell}-c_{low,\ell})\prod_{\ell\in S_j/\{k\}}\min(c_{high,\ell},\gamma_\ell)}{\mu(R_t)}\\
\ge&\frac{\prod_{\ell\in[p]/S_j}(c_{high,\ell}-c_{low,\ell})\prod_{\ell\in S_j/\{k\}}c_{high,\ell}\cdot\gamma_\ell}{\mu(R_t)}\\
=&\frac{\mu(R_t)\cdot\prod_{\ell\in S_j/\{k\}}\gamma_\ell}{\mu(R_t)}\\
\ge&C_\gamma^{s_j-1}.
\end{aligned}$$

163 That completes the proof. □

Lemma S9. *Suppose that constraint C4 from the main text holds. Then, for any fixed $\epsilon>0$ it holds true that*

$$P\left(\inf_{T\in\mathcal{T}_0}\ \min_{\substack{t\in T,\mu(R_t)\ge\epsilon,\\ \mathring{U}(t)\neq\emptyset}}\ \min_{k\in\mathring{U}(t)}\ \sup_{\gamma\in[C_\gamma,1-C_\gamma]}\Delta_I^n(R_{t,l}(\gamma;k),R_{t,r}(\gamma;k))>\frac{\epsilon}{4}C_\beta^2C_\gamma^{2\max_j s_j-1}\right)\to 1. \qquad [40]$$

Proof. First of all, we know from Proposition S6 that $\sup_{R_t\in\mathscr{R}}|\Delta_I^n(R_t)-\Delta_I(R_t)|\xrightarrow{p}0$. Thus, in order to prove Eq. (40), we only need to show that

$$\inf_{T\in\mathcal{T}_0}\ \min_{\substack{t\in T,\mu(R_t)\ge\epsilon,\\ \mathring{U}(t)\neq\emptyset}}\ \min_{k\in\mathring{U}(t)}\Delta_I(R_{t,l}(\gamma_k;k),R_{t,r}(\gamma_k;k))>\frac{\epsilon}{2}C_\beta^2C_\gamma^{2\max_j s_j-1}. \qquad [41]$$

Recall that γ_k is the ground-truth threshold of feature k in the interaction. Here we can drop $\max_{\gamma\in[C_\gamma,1-C_\gamma]}$ and use γ_k because that results in a lower bound of the previous equation. Based on Lemma S7, we know that

$$\begin{aligned}
&\Delta_I(R_{t,l}(\gamma_k;k),R_{t,r}(\gamma_k;k))\\
=&\mu(R_t)\cdot\beta_j^2P(\forall\ \ell\in S_j/\{k\},\ X_\ell\le\gamma_\ell|X\in R_t)^2\cdot(1-\gamma_k)\gamma_k\\
\ge&\frac{1}{2}C_\gamma C_\beta^2\epsilon\cdot P(\forall\ \ell\in S_j/\{k\},\ X_\ell\le\gamma_\ell|X\in R_t)^2.
\end{aligned}$$

164 The second inequality is due to $\mu(R_t)\ge\epsilon$, $\gamma_k(1-\gamma_k)\ge C_\gamma(1-C_\gamma)\ge\frac{1}{2}C_\gamma$ and $\beta_j\ge C_\beta$. Then using Lemma S8 leads to the
165 conclusion. □

For a node t, denote $\gamma^*_{t,k} = \mathrm{argmax}_{\gamma \in [C_\gamma, 1-C_\gamma]} \Delta^n_I(R_{t,l}(\gamma; k), R_{t,r}(\gamma; k))$.

Lemma S10. *Suppose that constraint C4 from the main text holds true, then we have*

$$\sup_{T \in \mathcal{T}_0} \max_{\substack{t \in T, \mu(R_t) \geq \epsilon, \\ \mathring{U}(t) \neq \emptyset}} \max_{k \in \mathring{U}(t)} |\gamma^*_{t,k} - \gamma_k| \xrightarrow{p} 0.$$

Proof. To simplify the notation in the proof, let us denote

$$\begin{aligned} a_n &= \Delta^n_I(R_{t,l}(\gamma^*_{t,k}; k), R_{t,r}(\gamma^*_{t,k}; k)), \\ a &= \Delta_I(R_{t,l}(\gamma^*_{t,k}; k), R_{t,r}(\gamma^*_{t,k}; k)) \\ b_n &= \Delta^n_I(R_{t,l}(\gamma_k; k), R_{t,r}(\gamma_k; k)), \\ b &= \Delta_I(R_{t,l}(\gamma_k; k), R_{t,r}(\gamma_k; k)). \end{aligned}$$

Using Proposition S6, we have

$$\sup_{T \in \mathcal{T}_0} \max_{\substack{t \in T, \mu(R_t) \geq \epsilon, \\ \mathring{U}(t) \neq \emptyset}} \max_{k \in \mathring{U}(t)} \Big| a_n - a \Big| \xrightarrow{p} 0. \qquad [42]$$

By Lemma S9 (see Eq. (41)), we know the second term is bounded uniformly above zero:

$$\inf_{T \in \mathcal{T}_0} \min_{\substack{t \in T, \mu(R_t) \geq \epsilon, \\ \mathring{U}(t) \neq \emptyset}} \min_{k \in \mathring{U}(t)} a \geq \frac{\epsilon}{2} C^2_\beta C_\gamma^{2 \max_j s_j - 1}.$$

Thus, the ratio converges to 1 in probability:

$$\sup_{T \in \mathcal{T}_0} \max_{\substack{t \in T, \mu(R_t) \geq \epsilon, \\ \mathring{U}(t) \neq \emptyset}} \max_{k \in \mathring{U}(t)} \Big| \frac{a_n}{a} - 1 \Big| \xrightarrow{p} 0. \qquad [43]$$

Similarly, this applies to b_n and b, i.e.,

$$\sup_{T \in \mathcal{T}_0} \max_{\substack{t \in T, \mu(R_t) \geq \epsilon, \\ \mathring{U}(t) \neq \emptyset}} \max_{k \in \mathring{U}(t)} \Big| \frac{b_n}{b} - 1 \Big| \xrightarrow{p} 0. \qquad [44]$$

So by the continuous mapping theorem, we know that

$$\sup_{T \in \mathcal{T}_0} \max_{\substack{t \in T, \mu(R_t) \geq \epsilon, \\ \mathring{U}(t) \neq \emptyset}} \max_{k \in \mathring{U}(t)} \Big| \frac{b_n}{a_n} \frac{a}{b} - 1 \Big| \xrightarrow{p} 0.$$

Because $\gamma^*_{t,k}$ maximizes Δ^n_I and γ_k maximizes Δ_I, $a_n \geq b_n$ and $a \leq b$. Thus $\frac{b_n}{a_n} \frac{a}{b} \leq \frac{a}{b} \leq 1$. Therefore, we know that

$$\sup_{T \in \mathcal{T}_0} \max_{\substack{t \in T, \mu(R_t) \geq \epsilon, \\ \mathring{U}(t) \neq \emptyset}} \max_{k \in \mathring{U}(t)} 1 - \frac{a}{b} \xrightarrow{p} 0.$$

By Lemma S7, we know that

$$\begin{aligned} a =& \mu(R_t) \cdot \beta_j^2 P(\forall\, \ell \in S_j/\{k\},\ X_\ell \leq \gamma_\ell | X \in R_t)^2 \cdot \Big(\mathbf{1}(\gamma^*_{t,k} \leq \gamma_k) \cdot \frac{(1-\gamma_k)^2 \gamma^*_{t,k}}{(1-\gamma^*_{t,k})} + \mathbf{1}(\gamma^*_{t,k} > \gamma_k) \cdot \frac{\gamma_k^2 (1-\gamma^*_{t,k})}{\gamma^*_{t,k}} \Big), \\ b =& \mu(R_t) \cdot \beta_j^2 P(\forall\, \ell \in S_j/\{k\},\ X_\ell \leq \gamma_\ell | X \in R_t)^2 \cdot \gamma_k(1-\gamma_k). \end{aligned}$$

Thus the ratio is

$$\frac{a}{b} = \mathbf{1}(\gamma^*_{t,k} \leq \gamma_k) \cdot \frac{(1-\gamma_k)\gamma^*_{t,k}}{\gamma_k(1-\gamma^*_{t,k})} + \mathbf{1}(\gamma^*_{t,k} > \gamma_k) \cdot \frac{\gamma_k(1-\gamma^*_{t,k})}{(1-\gamma_k)\gamma^*_{t,k}}.$$

When $\gamma^*_{t,k} \leq \gamma_k$,

$$\begin{aligned} 1 - \frac{a}{b} &= 1 - \frac{(1-\gamma_k)\gamma^*_{t,k}}{\gamma_k(1-\gamma^*_{t,k})} \\ &= \frac{\gamma_k - \gamma^*_{t,k}}{\gamma_k(1-\gamma^*_{t,k})} \geq \gamma_k - \gamma^*_{t,k}. \end{aligned}$$

Similarly, when $\gamma^*_{t,k} \geq \gamma_k$, then $1 - \frac{a}{b} \geq \gamma^*_{t,k} - \gamma_k$. Thus, $1 - a/b \geq |\gamma_k - \gamma^*_{t,k}| \geq 0$. Thus, by the Squeeze theorem, we complete the proof. ☐

Lemma S11. *Suppose that constraint C4 from the main text holds. Then the following statements are true:*

i) For any fixed $\epsilon, \delta > 0$,

$$P\left(\inf_{T \in \mathcal{T}_1(\mathcal{D})} \min_{\substack{t \in T, \mu(R_t) \geq \epsilon \\ U(t) \neq \emptyset}} \min_{j \in [J]} \min_{k \in S_j \cap U(t)} P(\forall\, \ell \in S_j/\{k\},\ X_\ell \leq \gamma_\ell | X \in R_t; \mathcal{D}) - C_\gamma^{s_j - 1} \geq -\delta\right) \to 1.$$

ii) For any fixed $\epsilon > 0$,

$$P\left(\inf_{T \in \mathcal{T}_1(\mathcal{D})} \min_{\substack{t \in T, \mu(R_t) \geq \epsilon, \\ U(t) \neq \emptyset}} \min_{k \in U(t)} \sup_{\gamma \in [C_\gamma, 1 - C_\gamma]} \Delta_I^n(R_{t,l}(\gamma; k), R_{t,r}(\gamma; k)) > \frac{\epsilon}{4} C_\beta^2 C_\gamma^{2 \max_j s_j - 1}\right) \to 1.$$

iii)

$$\sup_{T \in \mathcal{T}_1(\mathcal{D})} \max_{\substack{t \in T, \mu(R_t) \geq \epsilon, \\ U(t) \neq \emptyset}} \max_{k \in U(t)} |\gamma_{t,k}^* - \gamma_k| \xrightarrow{p} 0.$$

Proof. We use math induction to show that the above statements hold for any $L \geq 0$:

i) For any fixed $\epsilon, \delta > 0$,

$$P\left(\inf_{T \in \mathcal{T}_1(\mathcal{D})} \min_{\substack{t \in T, \mu(R_t) \geq \epsilon, U(t) \neq \emptyset, \\ \sum_{j=1}^J |\dot{\mathrm{F}}^{\pm}(t) \cap S_j^+| \leq L}} \min_{j \in [J]} \min_{k \in S_j \cap U(t)} P(\forall\, \ell \in S_j/\{k\},\ X_\ell \leq \gamma_\ell | X \in R_t; \mathcal{D}) - C_\gamma^{s_j - 1} \geq -\delta\right) \to 1.$$

ii) For any fixed $\epsilon > 0$,

$$P\left(\inf_{T \in \mathcal{T}_1(\mathcal{D})} \min_{\substack{t \in T, \mu(R_t) \geq \epsilon, U(t) \neq \emptyset, \\ \sum_{j=1}^J |\dot{\mathrm{F}}^{\pm}(t) \cap S_j^+| \leq L}} \min_{k \in U(t)} \sup_{\gamma \in [C_\gamma, 1 - C_\gamma]} \Delta_I^n(R_{t,l}(\gamma; k), R_{t,r}(\gamma; k)) > \frac{\epsilon}{4} C_\beta^2 C_\gamma^{2 \max_j s_j - 1}\right) \to 1.$$

iii)

$$\sup_{T \in \mathcal{T}_1(\mathcal{D})} \max_{\substack{t \in T, \mu(R_t) \geq \epsilon, U(t) \neq \emptyset, \\ \sum_{j=1}^J |\dot{\mathrm{F}}^{\pm}(t) \cap S_j^+| \leq L}} \max_{k \in U(t)} |\gamma_{t,k}^* - \gamma_k| \xrightarrow{p} 0.$$

If those statements are true, then our proof is complete because for any node t, $\sum_{j=1}^J |\dot{\mathrm{F}}^{\pm}(t) \cap S_j^+| \leq \sum_j s_j$, which is a constant.

When $L = 0$, $U(t) \neq \emptyset$ and $\sum_j |\dot{\mathrm{F}}^{\pm}(t) \cap S_j^+| = 0$ implies that $U(t) = \cup_{j=1}^J S_j / F(t) = \mathring{U}(t) \neq \emptyset$. Then the statement holds because of Lemmas S8, S9, and S10.

Suppose the statement holds for $L = L_0$, and let us consider the case $L = L_0 + 1$:

i): For $k \in S_j \cap U(t)$, we know that $S_j^+ \cap \mathrm{F}^{\pm}(t) = \emptyset$. Now consider $S_j^+ \cap \dot{\mathrm{F}}^{\pm}(t)$: if it is also empty, then $k \in \mathring{U}(t)$ and i) holds because of Lemma S8. Let's consider the case when $S_j^+ \cap \dot{\mathrm{F}}^{\pm}(t) \neq \emptyset$. For any $\ell \in S_j^+ \cap \dot{\mathrm{F}}^{\pm}(t)$, some parent nodes of t are split on feature ℓ and node t is at the left branch of the first parent node that is split on ℓ. In other words, this is the scenario where $(\ell, -1)$ first appears in the path and then $(\ell, +1)$ appears later. Denote that first parent node that is split on ℓ to be $t_{parent,\ell}$. Since none of $t_{parent,\ell}$'s parent nodes are split on ℓ, $\ell \in S_j^+ \cap \dot{\mathrm{F}}^{\pm}(t)$ but not in $S_j^+ \cap \dot{\mathrm{F}}^{\pm}(t_{parent,\ell})$. Since $\dot{\mathrm{F}}^{\pm}(t_{parent,\ell})$

is a subset of $\dot{\mathrm{F}}^{\pm}(t)$, we know that $\sum_{j=1}^{J} |S_j^+ \cap \dot{\mathrm{F}}^{\pm}(t_{parent,\ell})| \leq L_0$. Also, because $S_j^+ \cap \dot{\mathrm{F}}^{\pm}(t_{parent,\ell}) = \emptyset$ and $\ell \notin \dot{F}(t_{parent,\ell})$, we know that $\ell \in U(t_{parent,\ell})$. Then by the induction condition iii), we know that $\gamma^*_{t_{parent,\ell},\ell} \xrightarrow{p} \gamma_\ell$. Because t is at the left branch of $t_{parent,\ell}$, the upper bound in R_t for feature ℓ, i.e., $c_{high,\ell}$, is smaller or equal to $\gamma^*_{t_{parent,\ell},\ell}$. In other words, for any fixed $\delta > 0$, we know that

$$P\left(\sup_{T\in\mathcal{T}_1(\mathcal{D})} \max_{\substack{t\in T, \mu(R_t)\geq\epsilon, U(t)\neq\emptyset,\\ \sum_{j=1}^{J}|\dot{\mathrm{F}}^{\pm}(t)\cap S_j^+|\leq L_0+1}} \max_{\substack{j\in[J]\\ S_j^+\cap \mathrm{F}^{\pm}(t)=\emptyset}} \max_{(\ell,+1)\in S_j^+\cap\dot{\mathrm{F}}^{\pm}(t)} c_{high,\ell} - \gamma_\ell > \delta\right) \xrightarrow{p} 0.$$

For any l such that $\ell \in S_j$ but $(\ell, +1) \notin S_j^+ \cap \dot{\mathrm{F}}^{\pm}(t)$, we have that $c_{low,\ell} = 0$. Note that $c_{high,k} = 1$ and $c_{low,k} = 0$ because $k \in U(t)$. Then, $P(\forall\, \ell \in S_j/\{k\},\ X_\ell \leq \gamma_\ell | X \in R_t; \mathcal{D})$ is

$$\begin{aligned}
&\frac{P(\forall \ell \in S_j/\{k\}\ X_\ell \leq \gamma_\ell, X \in R_t; \mathcal{D})}{\mu(R_t)}\\
=&\frac{\prod_{\ell\in[p]/S_j}(c_{high,\ell}-c_{low,\ell})\prod_{\ell\in S_j/\{k\}}\max(\min(c_{high,\ell},\gamma_\ell)-c_{low,\ell},0)}{\mu(R_t)}\\
=&\frac{\prod_{\ell\in[p]/S_j}(c_{high,\ell}-c_{low,\ell})\prod_{(\ell,+1)\in S_j^+\cap\dot{\mathrm{F}}^{\pm}(t)}(c_{high,\ell}-c_{low,\ell}+o_p(1))\prod_{\ell\in S_j/\{k\},(\ell,+1)\notin S_j^+\cap\dot{\mathrm{F}}^{\pm}(t)}\min(c_{high,\ell},\gamma_\ell)}{\mu(R_t)}\\
\geq&\frac{\prod_{\ell\in[p]/S_j}(c_{high,\ell}-c_{low,\ell})\prod_{(\ell,+1)\in S_j^+\cap\dot{\mathrm{F}}^{\pm}(t)}(c_{high,\ell}-c_{low,\ell})\prod_{\ell\in S_j/\{k\},(\ell,+1)\notin S_j^+\cap\dot{\mathrm{F}}^{\pm}(t)}c_{high,\ell}\cdot\gamma_\ell}{\mu(R_t)}+o_p(1)\\
\geq&\frac{\mu(R_t)\cdot\prod_{\ell\in S_j/\{k\},(\ell,+1)\notin S_j^+\cap\dot{\mathrm{F}}^{\pm}(t)}\gamma_\ell}{\mu(R_t)}+o_p(1)\\
\geq&C_\gamma^{s_j-1}+o_p(1),
\end{aligned}$$

where the first equality follows from Eq. (39). That completes the proof for i).

ii): Given i), ii) follows analog as in the proof of Lemma S9.

iii) Given ii), iii) follows analog as in the proof of Lemma S10.

Thus, we have finished the math induction and proved the statements. □

Lemma S12. *For any tree $T \in \mathcal{T}_1$ and any node $t \in T$, the noisy features correspond to a nearly zero impurity decrease, i.e.*

$$\sup_{T\in\mathcal{T}_1}\max_{t\in T}\max_{k\in[p]/U(t)}\sup_{\gamma\in[0,1]}\Delta_I^n(R_{t,l}(\gamma;k),R_{t,r}(\gamma;k))\xrightarrow{p}0. \quad [45]$$

Proof. By Proposition S6, we only need to show that

$$\sup_{T\in\mathcal{T}_1}\max_{t\in T}\max_{k\in[p]/U(t)}\sup_{\gamma\in[0,1]}\Delta_I(R_{t,l}(\gamma;k),R_{t,r}(\gamma;k))\xrightarrow{p}0. \quad [46]$$

For $k \in [p]/U(t)$, either $k \in [p]/\bigcup_{j=1}^{J} S_j$ or $k \in \bigcup_{j=1}^{J} S_j/U(t)$. We will analyze these two cases separately:

First, assume that $k \in [p]/\bigcup_{j=1}^{J} S_j$. For any $j' \in [J]$, it follows that k is not contained in $S_{j'}$. Because different features are independent, X_k is independent from $X \in \{X | \forall\, \ell \in S_{j'},\ X_\ell \leq \gamma_\ell\}$. Therefore, for any $j' \in [J]$, we have

$$P(\forall\, \ell \in S_{j'},\ X_\ell \leq \gamma_\ell | X \in R_{t,l}(\gamma;k)) = P(\forall\, \ell \in S_{j'},\ X_\ell \leq \gamma_\ell | X \in R_{t,r}(\gamma;k)).$$

That implies $\Delta_I(R_{t,l}(\gamma;k), R_{t,r}(\gamma;k)) = 0$.

Second, assume that there exists j such that $k \in S_j/U(t)$. For $j' \neq j$, by a similar deduction as before, we know that

$$P(\forall\, \ell \in S_{j'},\ X_\ell \leq \gamma_\ell | X \in R_{t,l}(\gamma;k)) = P(\forall\, \ell \in S_{j'},\ X_\ell \leq \gamma_\ell | X \in R_{t,r}(\gamma;k)).$$

The impurity decrease $\Delta_I(R_{t,l}(\gamma;k), R_{t,r}(\gamma;k))$ becomes

$$\frac{\mu(R_{t,l}(\gamma;k))\mu(R_{t,r}(\gamma;k))}{\mu(R_t)}\beta_j^2\Big(P(\forall\,\ell\in S_j,\ X_\ell\leq\gamma_\ell|X\in R_{t,l}(\gamma;k))-P(\forall\,\ell\in S_j,\ X_\ell\leq\gamma_\ell|X\in R_{t,r}(\gamma;k))\Big)^2. \quad [47]$$

Again, we consider two cases: Because $k \notin U(t)$, either $(k, -1) \in \mathrm{F}^{\pm}(t)$ or $S_j^+ \cap \mathrm{F}^{\pm}(t) \neq \emptyset$.

i) If $S_j^+ \cap \mathrm{F}^{\pm}(t) \neq \emptyset$, suppose $(k', +1)$ is the first positive signed feature in S_j^+ that enters $\mathrm{F}^{\pm}(t)$. That means we can find a parent of t, denoted as t_{parent}, that splits on feature k' and none of t_{parent}'s parent splits on k'. That implies $k' \notin \mathrm{F}(t_{parent})$ and $S_j^+ \cap \mathrm{F}^{\pm}(t_{parent}) = \emptyset$, in other words, $k' \in U(t_{parent})$. Recall that $\gamma^*_{t_{parent},k'}$ denotes the threshold at node t_{parent}. By

Lemma S11, we know that the threshold $\gamma^*_{t_{parent},k'} \xrightarrow{p} \gamma_{k'}$. Since t is on the right branch of the node t_{parent}, we have that $c_{low,k'}(t) \geq \gamma^*_{t_{parent},k'}$. Thus,

$$\mu(\{X|\forall\ \ell \in S_j,\ X_\ell \leq \gamma_\ell\} \cap R_t) \xrightarrow{p} 0.$$

Since Eq. (47) is bounded by

$$2C_\beta^2 \frac{\mu(R_{t,l}(\gamma;k))\mu(R_{t,r}(\gamma;k))}{\mu(R_t)}\Big[P(\forall\ \ell \in S_j,\ X_\ell \leq \gamma_\ell | X \in R_{t,l}(\gamma;k)) + P(\forall\ \ell \in S_j,\ X_\ell \leq \gamma_\ell | X \in R_{t,r}(\gamma;k))\Big]$$
$$\leq 2C_\beta^2 P(\forall\ \ell \in S_j,\ X_\ell \leq \gamma_\ell, X \in R_t),$$

we know that Eq. (47) converges to zero in probability.

ii) If $S_j^+ \cap \mathrm{F}^\pm(t) = \emptyset$ but $(k,-1) \in \mathrm{F}^\pm(t)$, it means there exists a parent of t, denoted t_{parent}, such that feature k is used to split that node and none of its parents splits on k, in other words, $k \in U(t_{parent})$. By Lemma S11, we know that the corresponding threshold $\gamma^*_{t_{parent},k} \xrightarrow{p} \gamma_k$. Since $S_j^+ \cap \mathrm{F}^\pm(t) = \emptyset$, it follows that t is on the left branch of t_{parent}. Thus, we have that $c_{high,k}(t) \leq \gamma^*_{t_{parent},k}$. For any fixed $\epsilon > 0$, if $\mu(R_{t,l}(\gamma;k)) > \epsilon$ and $\mu(R_{t,r}(\gamma;k)) > \epsilon$, then

$$P(\forall\ \ell \in S_j,\ X_\ell \leq \gamma_{\ell,j} | X \in R_{t,l}(\gamma;k)) - P(\forall\ \ell \in S_j,\ X_\ell \leq \gamma_{\ell,j} | X \in R_{t,r}(\gamma;k)) \xrightarrow{p} 0,$$

which implies that $\Delta_I(R_{t,l}(\gamma;k), R_{t,r}(\gamma;k)) \xrightarrow{p} 0$. Otherwise, $[\mu(R_{t,l}(\gamma;k)) \leq \epsilon$ or $\mu(R_{t,r}(\gamma;k)) \leq \epsilon$, and thus,

$$\frac{\mu(R_{t,l}(\gamma;k))\mu(R_{t,r}(\gamma;k))}{\mu(R_t)} \leq \epsilon$$

and

$$\Delta_I(R_{t,l}(\gamma;k), R_{t,r}(\gamma;k)) \leq 4\epsilon.$$

Since ϵ is chosen arbitrarily, this implies $\Delta_I(R_{t,l}(\gamma;k), R_{t,r}(\gamma;k)) \xrightarrow{p} 0$.

Combining a) and b), we complete the proof.

□

With the help of the previous lemmas, we have the following proposition:

Proposition S13. *Suppose t_{leaf} is a leaf of $\mathcal{P}$ from a random tree $T \in \mathcal{T}_2$. Suppose that constraint C4 and assumptions A1-A4 from the main text hold true. For any fixed constant $\epsilon > 0$, the following holds true:*

i)

$$P\left(\max_{t\in T}\max_{k\in[p]/U(t)} \Delta_I^n(R_{t,l}(\gamma^*_{t,k};k), R_{t,r}(\gamma^*_{t,k};k)) < \frac{\epsilon}{4}C_\beta^2 C_\gamma^{2\max_j s_j - 1}\right) \to 1.$$

ii)

$$P\left(U(t_{\mathrm{leaf}}) = \emptyset \middle| \mathcal{D}\right) \xrightarrow{p} 1.$$

iii)

$$P\left(\min_{t\in\mathfrak{p}(t_{\mathrm{leaf}})}\min_{k\in U(t)} \Delta_I^n(R_{t,l}(\gamma^*_{t,k};k), R_{t,r}(\gamma^*_{t,k};k)) \geq \frac{\epsilon}{4}C_\beta^2 C_\gamma^{2\max_j s_j - 1}\middle|\mathcal{D}\right) \geq 1 - \epsilon^{\tilde{C}} - \eta_n(\mathcal{D},\epsilon),$$

with constant $\tilde{C} = C_m^{2s}/\log(1/C_\gamma)$ and $\eta_n(\mathcal{D},\epsilon) \xrightarrow{p} 0$.

Proof. i) By Lemma S12, we know with probability approaching 1,

$$\max_{t\in T}\max_{k\in[p]/U(t)}\sup_{\gamma\in[0,1]} \Delta_I^n(R_{t,l}(\gamma;k), R_{t,r}(\gamma;k)) \leq \frac{\epsilon}{4}C_\beta^2 C_\gamma^{2\max_j s_j - 1}. \qquad [48]$$

ii): For any fixed $\epsilon > 0$, by Lemma S9 and Lemma S12, the following event A_ϵ happens with probability approaching 1,

$$A_\epsilon = \bigcap_{T\in\mathcal{T}_1}\Bigg\{\min_{t\in T, \mu(R_t)\geq\epsilon, U(t)\neq\emptyset}\ \min_{k\in U(t)}\ \sup_{\gamma\in[C_\gamma, 1-C_\gamma]} \Delta_I^n(R_{t,l}(\gamma;k)\}, R_{t,r}(\gamma;k)) > \max_{t\in T}\max_{k\in[p]/U(t)}\sup_{\gamma\in[0,1]} \Delta_I^n(R_{t,l}(\gamma;k), R_{t,r}(\gamma;k))\Bigg\}, \qquad [49]$$

which implies that for any node with volume at least ϵ any desirable features has higher impurity decrease than any non-desirable feature. For a random path $\mathcal{P}$, denote its leaf node t_{leaf} and the depth of the path is D. Then for $d \in [D]$, denote t_d to be the

d-th node on the path $\mathcal{P}(t_{\text{leaf}})$. Recall that $S = \cup_{j=1}^J S_j$ denotes the set of all signal features and $s = |S|$ their total number. Based on Eq. (49), if at any node t, its candidate feature set $M_{\text{try}}(t)$ contains all the signal features S, then it will split on a signal feature as long as $U(t_{\text{leaf}}) \neq \emptyset$. If there are more than s nodes along the path that has volume larger than ϵ and their candidate feature set contains S, then the desirable features must have been exhausted at the leaf node, i.e.,

$$\left\{ \left| \{d \in [D] : S \subset M_{\text{try}}(t_d) \text{ and } \mu(R_d) \geq \epsilon\} \right| \geq s, A_\epsilon \right\} \subset \{U(t_{\text{leaf}}) = \emptyset, A_\epsilon\}. \qquad [50]$$

Further, note that, because $\mu(R_{t_d}) \geq C_\gamma \mu(R_{t_{d-1}}) \geq \ldots \geq C_\gamma^d$, when $d < \log \epsilon / \log C_\gamma$, it always holds that $\mu(R_{t_{d-1}}) \geq \epsilon$ and therefore

$$\left\{ \left| \{d \in [\log \epsilon / \log C_\gamma] : S \subset M_{\text{try}}(t_d)\} \right| \geq s, A_\epsilon, D \geq \log \epsilon / \log C_\gamma \right\} \subset \{U(t_{\text{leaf}}) = \emptyset, A_\epsilon, D \geq \log \epsilon / \log C_\gamma\}. \qquad [51]$$

Since for any node t, its candidate feature set $M_{\text{try}}(t)$ has m_{try} features, we know

$$P(S \subset M_{\text{try}}(t)) = \frac{\binom{p-s}{m_{\text{try}}-s}}{\binom{p}{m_{\text{try}}}} = \frac{m_{\text{try}} \cdot (m_{\text{try}} - 1) \cdots (m_{\text{try}} - s + 1)}{p \cdot (p-1) \cdots (p-s+1)} \geq \left(\frac{m_{\text{try}} - s + 1}{p - s + 1} \right)^s \geq [C_m]^s.$$

Since $M_{\text{try}}(t)$ is independent of the path $\mathcal{P}$, it follows that

$$\begin{aligned} & P_{(\mathcal{P},T)} \left(\left| \{d \in [\log \epsilon / \log C_\gamma] : S \subset M_{\text{try}}(t_d)\} \right| \geq s \middle| D \geq \log \epsilon / \log C_\gamma, \mathcal{D} \right) \\ \geq\; & P(B(\log \epsilon / \log C_\gamma, [C_m]^s) \geq s) - \mathbf{1}(\mathcal{D} \in A_\epsilon) \\ \geq\; & 1 - \exp\left(-2 \log \epsilon / \log C_\gamma \left([C_m]^s - \frac{s}{\log \epsilon / \log C_\gamma} \right)^2 \right) - \mathbf{1}(\mathcal{D} \in A_\epsilon) \end{aligned}$$

where $B(n, p)$ denotes a Binomial distribution with n trails and success probability p and the last inequaility follows from Hoeffding's inequality. Thus, for any $0 < \epsilon < \exp((1 - 1/\sqrt{2})[C_m]^s / (s \log(1/C_\gamma)))$, we have

$$\left([C_m]^s - \frac{s}{\log \epsilon / \log C_\gamma} \right)^2 \geq \frac{1}{2} C_m^{2s}.$$

Denote

$$\tilde{C} = C_m^{2s} / \log(1/C_\gamma),$$

we have that for sufficiently large n

$$P_{(\mathcal{P},T)} \left(\left| \{d \in [\log \epsilon / \log C_\gamma] : S \subset M_{\text{try}}(t_d)\} \right| \geq s \middle| D(\mathcal{P}) \geq \log \epsilon / \log C_\gamma, \mathcal{D} \right) \geq 1 - \epsilon^{\tilde{C}} - \mathbf{1}(\mathcal{D} \in A_\epsilon) \qquad [52]$$

and thus it follows from Eq. (51) that

$$P_{(\mathcal{P},T)} \left(U(t_{\text{leaf}}) = \emptyset \middle| D \geq \log \epsilon / \log C_\gamma, \mathcal{D} \right) \geq 1 - \epsilon^{\tilde{C}} - \mathbf{1}(\mathcal{D} \in A_\epsilon). \qquad [53]$$

Because $P(D \geq \log \epsilon / \log C_\gamma) \to 1$, by the Markov inequality, we know the random variable

$$P(D \geq \log \epsilon / \log C_\gamma \middle| \mathcal{D}) \xrightarrow{p} 1.$$

Thus, we know

$$P_{(\mathcal{P},T)} \left(U(t_{\text{leaf}}) = \emptyset \middle| \mathcal{D} \right) \geq 1 - \epsilon^{\tilde{C}} + \eta_n(\mathcal{D}, \epsilon), \qquad [54]$$

where $\eta_n(\mathcal{D}, \epsilon)$ is a random variable only depend on $\mathcal{D}$ and $\eta_n(\mathcal{D}, \epsilon) \xrightarrow{p} 0$. Because that holds for any ϵ, we have

$$P_{(\mathcal{P},T)} \left(U(t_{\text{leaf}}) = \emptyset \middle| \mathcal{D} \right) \xrightarrow{p} 1.$$

iii) Denote t_s to be the s-th node in a path $\mathcal{P}(t_{\text{leaf}})$ for $s \geq 1$. Based on the proof of ii), let d be an integer that (roughly) equals to $\frac{\log \epsilon}{\log C_\gamma}$. Then $\mu(R_{t_d}) \geq \epsilon$ and $P(U(t_d) = \emptyset \middle| \mathcal{D}) \geq 1 - \epsilon^{\tilde{C}} + \eta_n(\mathcal{D}, \epsilon)$. When $U(t_d) = \emptyset$, it follows that $U(t_s) \neq \emptyset$ implies $s \leq d$ and $\mu(R_{t_s}) \geq \epsilon$. Thus,

$$P(\exists t \in \mathcal{P}(t_{\text{leaf}}), \text{ such that } U(t) \neq \emptyset \text{ and } \mu(R_t) < \epsilon | \mathcal{D}) \leq P(U(t_d) \neq \emptyset | \mathcal{D}) \leq \epsilon^{\tilde{C}} - \eta_n(\mathcal{D}, \epsilon).$$

Therefore, we have

$$\begin{aligned} & P\left(\min_{t \in \mathfrak{p}(t_{\text{leaf}}), U(t) \neq \emptyset} \min_{k \in U(t)} \Delta_I^n(R_{t,l}(\gamma_{t,k}^*; k), R_{t,r}(\gamma_{t,k}^*; k)) \geq \frac{\epsilon}{4} C_\beta^2 C_\gamma^{2 \max_j s_j - 1} \middle| \mathcal{D} \right) \\ \geq & P\left(\min_{t \in \mathfrak{p}(t_{\text{leaf}}), \mu(R_t) \geq \epsilon, U(t) \neq \emptyset} \min_{k \in U(t)} \Delta_I^n(R_{t,l}(\gamma_{t,k}^*; k), R_{t,r}(\gamma_{t,k}^*; k)) \geq \frac{\epsilon}{4} C_\beta^2 C_\gamma^{2 \max_j s_j - 1} \middle| \mathcal{D} \right) - \epsilon^{\tilde{C}} - \eta_n(\mathcal{D}, \epsilon), \end{aligned} \qquad [55]$$

thus, the proof follows from Lemma S11. □

B.2. Balanced root feature selection . Recall the definition of $C_{\text{root}}(\mathcal{D})$ in Eq. (7), which appears in Theorem S1. Recall that for any tree T from RF, there are two different sources of randomness: first, the randomness of the data $\mathcal{D} = ((\mathbf{x}_i, y_i))_{i=1}^n$, which we denoted as $(\mathcal{D})$, and second, the randomness from the candidate feature selection, which we denoted as (T). Denote $M_{\text{try}}(t) \subset [p]$ to be the set of candidate features selected at node t and note that $M_{\text{try}}(t)$ and the data $\mathcal{D}$ are independent.

Define the event A to be that, given data $\mathcal{D}$, the maximum impurity decrease at the split of root node for every signal feature $k \in \cup_j S_j$ is larger than that of any noisy feature $k' \notin \cup_j S_j$, that is,

$$A = \Big\{ \min_{k \in \cup_j S_j} \Delta_I^n \left(R_{t_{\text{root}},l}(\gamma_k^\star, k), R_{t_{\text{root}},r}(\gamma_k^\star, k)\right) > \max_{k' \notin \cup_j S_j} \Delta_I^n \left(R_{t_{\text{root}},l}(\gamma_{k'}^\star, k'), R_{t_{\text{root}},r}(\gamma_{k'}^\star, k')\right) \Big\}. \qquad [56]$$

Note that the event only depends on the data randomness $\mathcal{D}$ (and not on the tree randomness T and the path randomness $\mathcal{P}$). Thus, A is independent of $M_{\text{try}}(t_{\text{root}})$. Note that it follows from Proposition S13 that

$$P_{\mathcal{D}}(A) \to 1 \quad \text{as } n \to \infty.$$

Theorem S2. *Assume that $C_m p \le m_{try} \le (1-C_m)(p-s+1)+1$ for some constant $C_m \in (0,1)$. Condition on $\mathcal{D} = ((\mathbf{x}_i, y_i))_{i=1}^n$, for any $k \in \cup_j S_j$, we have that*

$$P_T\left(t_{\text{root}} \textit{ splits on feature } k |\, \mathcal{D}\right) \ge C_m^s - 1_{A^c}$$

and thus,

$$C_{\text{root}}(\mathcal{D}) \ge C_m^s - 1_{A^c} \xrightarrow{p} [C_m]^s \quad \textit{as } n \to \infty.$$

Proof. For any $k \in \cup_j S_j$, define B_k to be the event that only signal feature k is selected in $M_{\text{try}}(t_{\text{root}})$, that is,

$$B_k \triangleq \{M_{\text{try}}(t_{\text{root}}) \cap \cup_j S_j = k \text{ and } |M_{\text{try}}(t_{\text{root}}) \setminus \cup_j S_j| = m_{\text{try}} - 1\}.$$

Note that B_k only depends on $M_{\text{try}}(t_{\text{root}})$ but not on $\mathcal{D}$ and

$$A \cap B_k \subset \{t_{\text{root}} \text{ splits on feature } k\}.$$

Thus,

$$P_T\left(t_{\text{root}} \text{ splits on feature } k|\mathcal{D}\right) \ge P_T(B_k \cap A|\mathcal{D}) \ge P_T\left(B_k|\mathcal{D}\right) - P_T\left(A^c|\mathcal{D}\right) = P\left(B_k\right) - 1_{A^c}.$$

Moreover, we have that

$$\begin{aligned} P(B_k) &= \frac{\binom{p-s}{m_{\text{try}}-1}}{\binom{p}{m_{\text{try}}}} = \frac{m_{\text{try}}}{p} \frac{\binom{p-s}{m_{\text{try}}-1}}{\binom{p-1}{m_{\text{try}}-1}} = \frac{m_{\text{try}}}{p} \frac{\binom{p-m_{\text{try}}}{s-1}}{\binom{p-1}{s-1}} \\ &= \prod_{i=0}^{s-2} \left(\frac{p - m_{\text{try}} - i}{p-1-i}\right) \frac{m_{\text{try}}}{p} \ge \left(\frac{p - m_{\text{try}} - s + 2}{p - s + 1}\right)^{s-1} \frac{m_{\text{try}}}{p} \ge C_m^s, \end{aligned}$$

where the second equality follows from the identity

$$\frac{\binom{n-h}{k}}{\binom{n}{k}} = \frac{\binom{n-k}{h}}{\binom{n}{h}},$$

with where $n = p-1$, $h = s-1$, and $k = m_{\text{try}} - 1$. □

B.3. Combining results. Our major result in Theorem S1 is formulated for the random (oracle) feature set $\mathcal{F} = \mathcal{F}(\mathcal{D}, T, \mathcal{P})$. Note that this is an oracle feature set, as it depends on the true interactions S_j, which are not known in practice. From the analysis in Section B.1 we know that we can obtain a consistent estimate of the oracle feature set $\mathcal{F}$ by thresholding on MDI as in $\hat{\mathcal{F}}_\epsilon$. Recall that for a given ϵ the (random) set $\hat{\mathcal{F}}_\epsilon$ can easily be obtained without any knowledge of the true model. Based on Proposition S13, we observe the following.

Recall that Ω_0 is defined in Eq. (5), $\mathcal{F}$ is defined in Eq. (4), and $\hat{\mathcal{F}}_\epsilon$ is defined in Eq. (7) in the main text. We have the following theorem.

Theorem S3. *Under the assumption of Proposition S13 it holds true that for any fixed $\epsilon > 0$,*

$$P_{(\mathcal{P},T)}\left(\Omega_0^c \mid \mathcal{D}\right) \xrightarrow{p} 0; \qquad [57]$$

$$P_{(\mathcal{P},T)}\left(\hat{\mathcal{F}}_\epsilon \not\subseteq \mathcal{F} \;\middle|\; \mathcal{D}\right) \xrightarrow{p} 0; \qquad [58]$$

$$P_{(\mathcal{P},T)}\left(\hat{\mathcal{F}}_\epsilon \neq \mathcal{F} \;\middle|\; \mathcal{D}\right) \le \left(\frac{4\epsilon}{C_\beta^2 C_\gamma^{2\max_j s_j - 1}}\right)^{\tilde{C}} + \eta_n(\mathcal{D}, \epsilon) \quad \textit{with } \eta_n(\mathcal{D}, \epsilon) \xrightarrow{p} 0; \qquad [59]$$

with $\tilde{C}$ as in Proposition S13.

Proof. Eq. (57) follows directly from Proposition S13 ii) and the definition of Ω_0 in Eq. (5).

To prove Eq. (58), one observes from Proposition S13 i) that for any $\epsilon > 0$, taking $\tilde{\epsilon} = \frac{4\epsilon}{C_\beta^2 C_\gamma^{2\max_j s_j - 1}}$, the following happens with probability converging to one (as $n \to \infty$)

$$\max_{t\in T} \max_{k\in[p]/U(t)} \Delta_I^n(R_{t,l}(\gamma_{t,k}^*;k), R_{t,r}(\gamma_{t,k}^*;k)) < \frac{\tilde{\epsilon}}{4} C_\beta^2 C_\gamma^{2\max_j s_j - 1} = \epsilon,$$

which implies that $\hat{\mathcal{F}}_\epsilon$ contains no irrelevant features. Thus,

$$\liminf_{n\to\infty} P_{(\mathcal{D},T,\mathcal{P})}\left(\hat{\mathcal{F}}_\epsilon \subseteq \mathcal{F}\right) = 1.$$

Then by Markov inequality, we know $P_{(\mathcal{P},T)}\left(\hat{\mathcal{F}}_\epsilon \nsubseteq \mathcal{F} \;\middle|\; \mathcal{D}\right) \xrightarrow{p} 0$.

To prove Eq. (59), we further note that by Proposition S13 iii),

$$P\left(\min_{t\in\mathcal{P}(t_{\text{leaf}})} \min_{k\in U(t)} \Delta_I^n(R_{t,l}(\gamma_{t,k}^*;k), R_{t,r}(\gamma_{t,k}^*;k)) \geq \epsilon \middle| \mathcal{D}\right) \geq 1 - \left(\frac{4\epsilon}{C_\beta^2 C_\gamma^{2\max_j s_j - 1}}\right)^{\tilde{C}} - \eta_n(\mathcal{D},\epsilon).$$

If

$$\min_{t\in\mathfrak{p}(t_{\text{leaf}})} \min_{k\in U(t)} \Delta_I^n(R_{t,l}(\gamma_{t,k}^*;k), R_{t,r}(\gamma_{t,k}^*;k)) \geq \epsilon$$

and

$$\max_{t\in T} \max_{k\in[p]/U(t)} \Delta_I^n(R_{t,l}(\gamma_{t,k}^*;k), R_{t,r}(\gamma_{t,k}^*;k)) < \epsilon,$$

we know $\hat{\mathcal{F}}_\epsilon = \mathcal{F}$. Thus, we have

$$P_{(T,\mathcal{P})}\left(\hat{\mathcal{F}}_\epsilon = \mathcal{F}\middle|\mathcal{D}\right) \geq 1 - \left(\frac{4\epsilon}{C_\beta^2 C_\gamma^{2\max_j s_j - 1}}\right)^{\tilde{C}} - \eta_n(\mathcal{D},\epsilon). \quad [60]$$

That completes the proof. □

Finally, we can combine Theorem S1, Theorem S2, and Theorem S3 to prove Theorem 1 and 2 in the main text.

Proof of Theorem 1. Assume that $|S^\pm| = \tilde{s}$ and $S^\pm = \{(k_1,b_1),\dots,(k_{\tilde{s}},b_{\tilde{s}})\}$. Analog as in Theorem S1, for any feature $k \in [p]$, let B^k be the Bernoulli random variable we draw when k appears for the first time on $\mathcal{P}$. Recall the definition of $\hat{\mathcal{F}}_\epsilon$, in particular, that $(k,b_k) \in \hat{\mathcal{F}}_\epsilon$ only if X_k appears the first time on $\mathcal{P}$. Thus, analog as for $\mathcal{F}$ (recall the proof of Theorem S1) we have that $(k,-1) \in \mathcal{F}$ implies $B^k = -1$ and $(k,+1) \in \mathcal{F}$ implies $B^k = +1$. Thus,

$$\{S^\pm \in \hat{\mathcal{F}}_\epsilon\} \subset \{B^{k_1} = b_1 \cap \ldots \cap B^{k_{\tilde{s}}} = b_{\tilde{s}}\}$$

and hence,

$$\text{DWP}(S^\pm) = P_{(\mathcal{P},T)}(S^\pm \in \hat{\mathcal{F}}_\epsilon|\mathcal{D}) \leq P_{(\mathcal{P},T)}(B^{k_1} = b_1 \cap \ldots \cap B^{k_{\tilde{s}}} = b_{\tilde{s}}|\mathcal{D}) = P_{\mathcal{P}}(B^{k_1} = b_1 \cap \ldots B^{k_{\tilde{s}}} = b_{\tilde{s}}) = 2^{-\tilde{s}}.$$

□

Proof of Theorem 2. Assume that $|S^\pm| = \tilde{s}$ and $S^\pm = \{(k_1,b_1),\dots,(k_{\tilde{s}},b_{\tilde{s}})\}$ and let

$$r_n(\mathcal{D},\epsilon) = \max\left(P_{(\mathcal{P},T)}(\Omega_0^c|\mathcal{D}) + \eta_n(\mathcal{D},\epsilon),\ P_{(\mathcal{P},T)}(\hat{\mathcal{F}}_\epsilon \nsubseteq \mathcal{F}|\mathcal{D})\right),$$

with $\eta_n(\mathcal{D},\epsilon)$ as in Theorem S3. It follows from Theorem S3 that $r_n(\mathcal{D},\epsilon) \xrightarrow{p} 0$ as $n \to \infty$.

Proof of (Interaction lower bound):

Assume that $S^\pm$ is a union interaction. Then we have that

$$\begin{aligned}
\text{DWP}(S^\pm) &= P_{(\mathcal{P},T)}(S^\pm \in \hat{\mathcal{F}}_\epsilon|\mathcal{D}) \\
&\geq P_{(\mathcal{P},T)}(S^\pm \in \mathcal{F}|\mathcal{D}) - P_{(\mathcal{P},T)}(\hat{\mathcal{F}}_\epsilon \neq \mathcal{F}|\mathcal{D}) \\
&\geq P_{(\mathcal{P},T)}(S^\pm \in \mathcal{F}|\mathcal{D}) - \left(\frac{4\epsilon}{C_\beta^2 C_\gamma^{2\max_j s_j - 1}}\right)^{\tilde{C}} - \eta_n(\mathcal{D},\epsilon) \\
&\geq 0.5^{\tilde{s}} - P_{(\mathcal{P},T)}(\Omega_0^c|\mathcal{D}) - \left(\frac{4\epsilon}{C_\beta^2 C_\gamma^{2\max_j s_j - 1}}\right)^{\tilde{C}} - \eta_n(\mathcal{D},\epsilon) \\
&\geq 0.5^{\tilde{s}} - \left(\frac{4\epsilon}{C_\beta^2 C_\gamma^{2\max_j s_j - 1}}\right)^{\tilde{C}} - r_n(\mathcal{D},\epsilon),
\end{aligned}$$

where the second inequality follows from Corollary S3 and the third inequality follows from Theorem S1.

Proof of (Non-interaction upper bound):

Assume that $S^{\pm}$ is not a union interaction. Then we have that

$$\begin{aligned}\mathrm{DWP}(S^{\pm}) &= P_{(\mathcal{P},T)}(S^{\pm} \in \hat{\mathcal{F}}_{\epsilon}|\mathcal{D}) \\ &\leq P_{(\mathcal{P},T)}(S^{\pm} \in \mathcal{F}|\mathcal{D}) + P_{(\mathcal{P},T)}(\hat{\mathcal{F}}_{\epsilon} \nsubseteq \mathcal{F}|\mathcal{D}) \\ &\leq 0.5^{\tilde{s}}(1 - C_{\mathrm{root}}(\mathcal{D})/2) + r_n(\mathcal{D}, \epsilon),\end{aligned}$$

where the second inequality follows from Theorem S2. □

S2. Additional figures

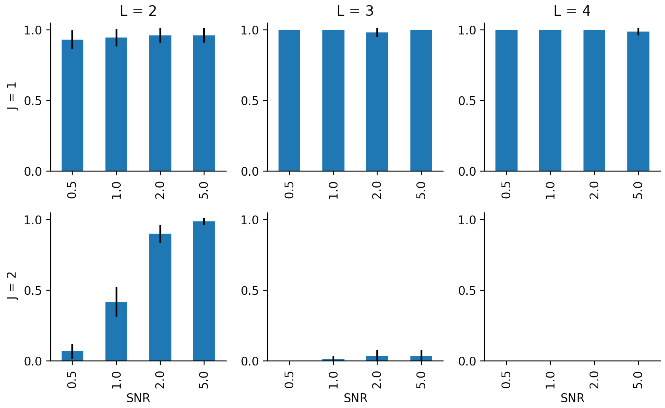

Fig. S1. Simulation results for the performance of the LSSFind (Algorithm 1). The data is generated from an LSS model with Gaussian noise as in Eq. (13) with $n = 1,000$ samples and $p = 20$ features. Standard deviations of the proximity scores are given in error bars. Different number of basic interactions K are shown in different rows, of interaction-orders L in different columns, and of a series of SNRs on the x-axis. The y-axis shows the proximity score in Eq. (14). A proximity score of one corresponds to perfect recovery of all interactions simultaneously.

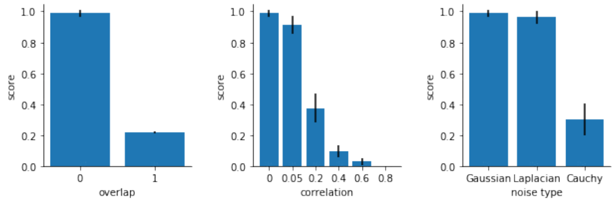

Fig. S2. Simulation results similar to those in Figure S1 for interactions of order $L = 2$ and $J = 2$, but when the data is generated from a mis-specified version of the LSS model, with $n = 1,000$ samples and $p = 20$ features. Left : signed features of different basic interactions are overlapping. When $\mathrm{overlap} = 1$, the basic interactions are $((1,-1),(2,-1)), ((2,-1),(3,-1))$. Middle: different features are correlated instead of independent. When corr $= \alpha$, the correlation between feature j_1 and j_2 is $\alpha^{|j_1-j_2|}$. Right: the noise follows a Laplace or Cauchy distribution, instead of Gaussian distributions.

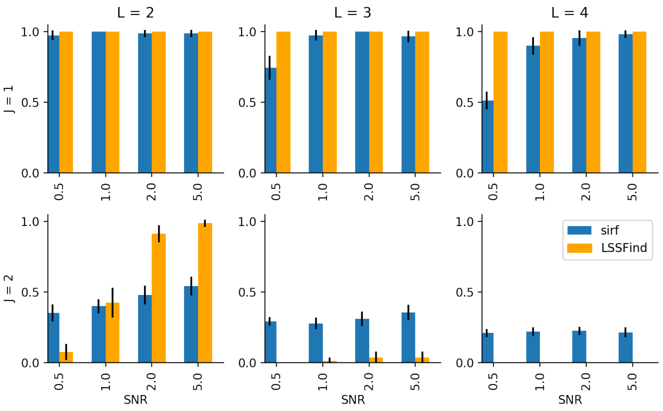

Fig. S3. Simulation results of LSSFind (orange) and iRF (blue) analog as in Figure S1 but with the performance measure $\mathrm{Eq.}\ (15)$ instead of $\mathrm{Eq.}\ (14)$.

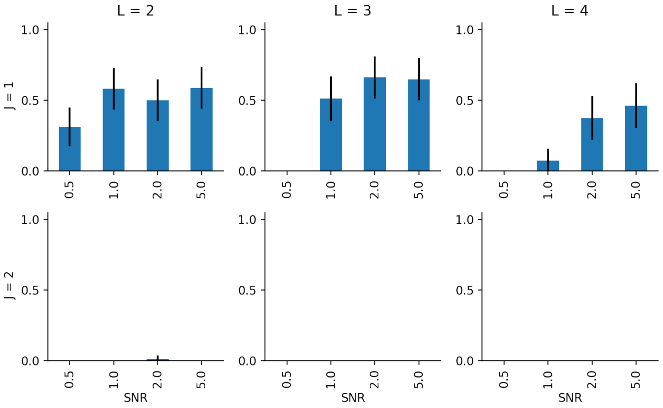

Fig. S4. Simulation results for iRF analog as in Figure S1. LSSFind has higher score when the number of basic interactions $K = 1$ or when the order of interactions $L = 2$. For other cases, neither methods have good performance. Note that when a different metric is used, the story is different, see Figure S3.

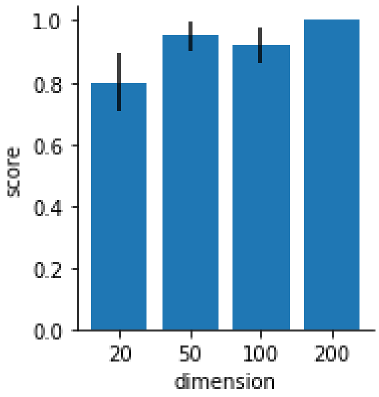

Fig. S5. Simulation results for LSSFind when the dimension p grows and the samplesize grows at the rate of $log(p)$. LSSFind has higher score when the dimension grows higher.

References

1. V Vapnik, Statistical learning theory (1998).
